# RNA G-quadruplex removal promotes a translational switch after meiosis resumption

**DOI:** 10.1093/nar/gkaf067

**Published:** 2025-04-30

**Authors:** Qiong-Wen Lu, Shao-Yuan Liu, Xiu-Quan Liao, Jing Chen, Zhi-Yan Jiang, Yu-Ke Wu, Heng-Yu Fan, Yu-Jing Lu, Qian-Qian Sha

**Affiliations:** GMU-GIBH Joint School of Life Sciences, The Guangdong-Hong Kong-Macau Joint Laboratory for Cell Fate Regulation and Diseases, Guangzhou Medical University, Guangzhou 510006, China; School of Biomedical and Pharmaceutical Sciences, Guangdong University of Technology, Guangzhou 510006, China; GMU-GIBH Joint School of Life Sciences, The Guangdong-Hong Kong-Macau Joint Laboratory for Cell Fate Regulation and Diseases, Guangzhou Medical University, Guangzhou 510006, China; GMU-GIBH Joint School of Life Sciences, The Guangdong-Hong Kong-Macau Joint Laboratory for Cell Fate Regulation and Diseases, Guangzhou Medical University, Guangzhou 510006, China; GMU-GIBH Joint School of Life Sciences, The Guangdong-Hong Kong-Macau Joint Laboratory for Cell Fate Regulation and Diseases, Guangzhou Medical University, Guangzhou 510006, China; MOE Key Laboratory for Biosystems Homeostasis and Protection and Innovation Center for Cell Signaling Network, Life Sciences Institute, Zhejiang University, Hangzhou 310058, China; MOE Key Laboratory for Biosystems Homeostasis and Protection and Innovation Center for Cell Signaling Network, Life Sciences Institute, Zhejiang University, Hangzhou 310058, China; MOE Key Laboratory for Biosystems Homeostasis and Protection and Innovation Center for Cell Signaling Network, Life Sciences Institute, Zhejiang University, Hangzhou 310058, China; School of Biomedical and Pharmaceutical Sciences, Guangdong University of Technology, Guangzhou 510006, China; Smart Medical Innovation Technology Center, Guangdong University of Technology, Guangzhou 510006, China; Guangdong Medicine-Engineering Interdisciplinary Technology Research Center, Guangzhou 510006, China; GMU-GIBH Joint School of Life Sciences, The Guangdong-Hong Kong-Macau Joint Laboratory for Cell Fate Regulation and Diseases, Guangzhou Medical University, Guangzhou 510006, China

## Abstract

Oocyte maturation-coupled mRNA post-transcriptional regulation is essential for the establishment of developmental potential. Previously, oocyte mRNA translation efficiencies focused on the trans-regulation of key RNA-binding protein (RBPs), rarely related to RNA structure. RNA G-quadruplexes (rG4s) are four-stranded RNA secondary structures involved in many different aspects of RNA metabolism. In this study, we have developed a low-input technique for rG4 detection (G4-LACE-seq) in mouse oocytes and found that rG4s were widely distributed in maternal transcripts, with enrichment in untranslated regions, and they underwent transcriptome-wide removal during meiotic maturation. The rG4-selective small-molecule ligand BYBX stabilized rG4s in the oocyte transcriptome and impaired spindle assembly and meiotic cell cycle progression. The proteomic spectrum results revealed that rG4 accumulation weakened the binding of a large number of RBPs to mRNAs, especially those associated with translational initiation. Ribosomal immunoprecipitation and translational reporter assays further proved that rG4s in the untranslated regions negatively affected the translational efficiency of key maternal mRNAs. Overexpression DEAH/RHA family helicase-36 partially reverses BYBX-induced oocyte developmental defects, suggesting its importance in rG4 regulation. Collectively, this study describes the distribution, dynamic changes, and regulation of rG4s in the mouse maternal transcriptome. Before meiosis resumption, a large number of rG4s in oocytes are necessary to maintain the translatome at a low level, and DHX36-mediated rG4 removal promotes a translational switch and is required for successful maternal-to-zygotic transition.

## Graphical Abstract

**Graphical Abstract:**
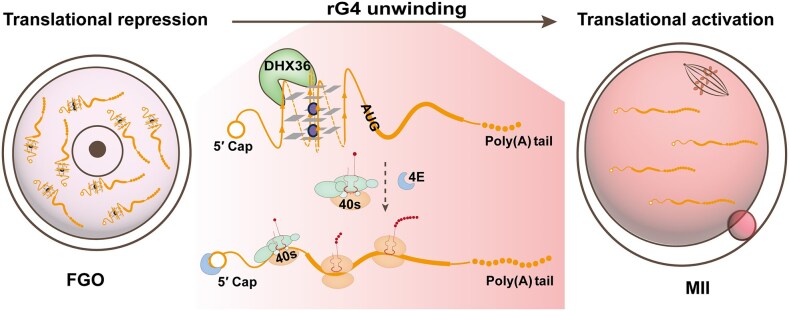

## Introduction

In mammalian species, including humans, the rapid growth of oocytes at the germinal vesicle (GV) stage is accompanied by rigorous transcription, but transcriptional activity is silenced in fully grown oocytes in the ovarian antral follicles, as well as in ovulated mature oocytes arrested at the metaphase II (MII) stage [1]. These maternal messenger RNA (mRNAs) remain stable and are translationally repressed [2–4]. Until transcriptional activity resumes during zygotic genome activation, oocytes depend completely on these maternal mRNAs to synthesize new proteins, ensuring meiotic maturation and subsequent fertilization [5]. To fulfill these functions, maternal mRNAs undergo polyadenylation, translational activation, and subsequent degradation during oocyte-to-embryo transition, also known as maternal-to-zygotic transition (MZT) [6, 7]. Therefore, mammalian oocyte maturation provides a unique and ideal model for studying post-transcriptional mRNA regulations [8].

RNAs are key regulators of almost every cellular process and the structures adopted by RNA molecules are thought to be central to their functions. Many RNAs are post-transcriptionally modified, and these modifications can stabilize or destabilize different RNA structures. For example, N6-methyladenosine modifications regulate RNA stability, translation, and degradation in mouse oocytes and early embryos, all of which are essential for proper embryonic development [9, 10]. N4-acetylcytidine modifications catalyzed by *N*-acetyltransferase 10 are required for mouse oocyte maturation as they facilitate mRNA translation and subsequent degradation [11–13]. Notably, special RNA structures, especially G-quadruplexes, are important for regulating RNA stability and translation in somatic cells [14, 15]. The RNA produced is abundant in oocytes, and it undergoes extensive post-transcriptional regulation during meiotic maturation. However, little is known about the dynamic changes and biomedical functions of mRNA structures in these processes.

G-quadruplexes are non-canonical higher-order secondary structures existing in both DNA (dG4s) and RNA G-quadruplexes (rG4s). They are formed through the self-recognition of guanines into stacked tetrads, in which the guanines are bonded by Hoogsteen hydrogen base-pairing [16] and stabilized by central monovalent cations such as K^+^ and Na^+^ [17]. rG4s have been found to be widely distributed in the nucleus, cytoplasm, and stress granules [18, 19] but have received less attention until recently compared to dG4s. Nevertheless, rG4s form more readily than dG4s *in vitro* owing to their increased thermodynamic stability and reduced steric hindrance [20]. Computational analyses and high-throughput investigations of the human transcriptome suggest that a large number of putative rG4s are present in mRNAs and are particularly enriched in 5′- and 3′-untranslated regions (UTRs) [21, 22]. It has been demonstrated that the rG4s in the 5′-UTR negatively affected the initiation and efficiency of translation [23, 24]. Evidence from chemical biology and genetics experiments also supports the role of rG4 in regard to the translational regulation of different mRNAs, such as NRAS [24], BCL-2 [25], and ADAR [26]. On the other hand, the rG4s in 3′-UTR are reported to regulate the mRNA transport and stability [27–29]. These previous studies were limited to pathological processes and *in vitro* cultured cell lines. The importance of rG4s in the developmental processes of specific cell types *in vivo* requires further investigation.

Helicases, including RecQ helicase [30–32], DEAD-box RNA helicase [33], DEAH-box helicase [34, 35], and Pif1 helicase [36], play important roles in regulating G4 dynamics by unwinding dG4s and rG4s in an ATPase-dependent manner. Among them, DHX36 is the most intensively studied and exhibits strong binding capacity and unfolding activity toward rG4s and positively regulates the translation of ADAR [26] and C9orf72 [37]. DHX36 is most abundant in the testes, and deficiency of DHX36 in spermatogonia leads to male infertility, suggesting that it has important functions in germ cells [38]. The knockout of certain RNA helicases, such as DDX3X, has been shown to lead to female infertility and premature ovarian failure [39]. During early embryonic development in zebrafish, the phase separation of DDX3X [40] helps to unravel the secondary structure of the 5′-UTR region of maternal mRNAs to which it binds, regulates the translation of these mRNAs, and affects maternal-zygotic transition and embryonic development. These findings validate the effects of RNA helicases on female fertility in different model organisms, highlighting the key role of RNA secondary structure in regulating fertility.

Recently, several G4 fluorescent probes have been developed as effective research tools. Although they have been shown to exhibit high specificity and affinity for G4s, most are unable to distinguish rG4s from dG4s. (Z)-3- (2-amino-2-oxoethyl)-1,1-dimethyl-2- ( (3-methylbenzo[d] thiazol-2 (3H)-ylidene) methyl)-1H-benzo[e]indol-3-ium bromide (BYBX) is a recently reported rG4 probe and stabilizer. BYBX specifically interacts with and stabilizes rG4s and simultaneously generates a fluorescence signal [41]. These characteristics of BYBX provide a new approach for evaluating the regulatory roles of rG4s in living cells.

Most classical experimental methods for rG4 detection, such as circular dichroism (CD) spectroscopy [42] and ultraviolet assay [43], are low-throughput and not applicable to genome-wide rG4 analyses. Researchers have developed high-throughput rG4 detection methods that can profile transcriptome-wide rG4s [21, 29, 44]. Unfortunately, these approaches require a total of 1 μg RNA, which is not applicable to low-input cells like oocytes. Linear amplification of complementary DNA ends and sequencing (LACE-seq) can be used to identify the targets of RNA-binding proteins (RBPs) by combining RBP-mediated reverse transcription termination with linear amplification and sequencing of complementary DNA ends, enabling the precise identification of RBP-binding sites at single-base resolution and at the single-cell level [45]. In the present study, we improved the LACE-seq method and established a low-input approach for rG4 detection in oocytes. We profiled transcriptome-wide rG4 dynamic changes during mouse oocyte maturation and investigated the impact of BYBX-induced rG4 stabilization in oocytes. Our study revealed that the rG4s accumulation on the 5′- or 3′-UTRs hindered the translational activation of key mRNAs, and highlighted the importance of rG4 removal on oocyte meiotic maturation.

## Materials and methods

### Animals

The mouse strains used in this study had a C57BL/6 genetic background. C57BL/6 mice were obtained from Daoke Medical & Pharmaceutical Company (Guangzhou, China). Mice were bred under specific pathogen-free conditions in a controlled environment of 20–22°C, with a 12/12 h light/dark cycle, 50–70% humidity, and food and water provided ad libitum. The experimental protocols used in this study were approved by the Institutional Animal Care and Use Committee of Guangzhou Medical University (GY2024-413) and Daoke Medical & Pharmaceutical Company (Guangzhou, China) (IACUC-DK-2023–06-10–01).

### Oocyte culture

The 4-week-old female mice were injected with 5 IU pregnant mare serum gonadotropin (PMSG; Ningbo Sansheng Pharmaceutical) and humanely euthanized 44h later. Oocytes were obtained in 37°C pre-warmed M2 medium (M7167; Sigma-Aldrich) at the GV stage and cultured in M16 medium (M7292; Sigma-Aldrich) covered with mineral oil (M5310; Sigma-Aldrich) at 37°C in a 5% CO_2_ atmosphere. BYBX (5 mM) was diluted to a gradient concentration using Dimethylsulfoxide (DMSO) and added to M16 medium. As a control, GV and MII oocytes were treated with the same concentration of DMSO. Milrinone (2 μM) was added to the culture medium to inhibit spontaneous germinal vesicle breakdown (GVBD).

### rG4-LACE-seq

**Figure 1.**
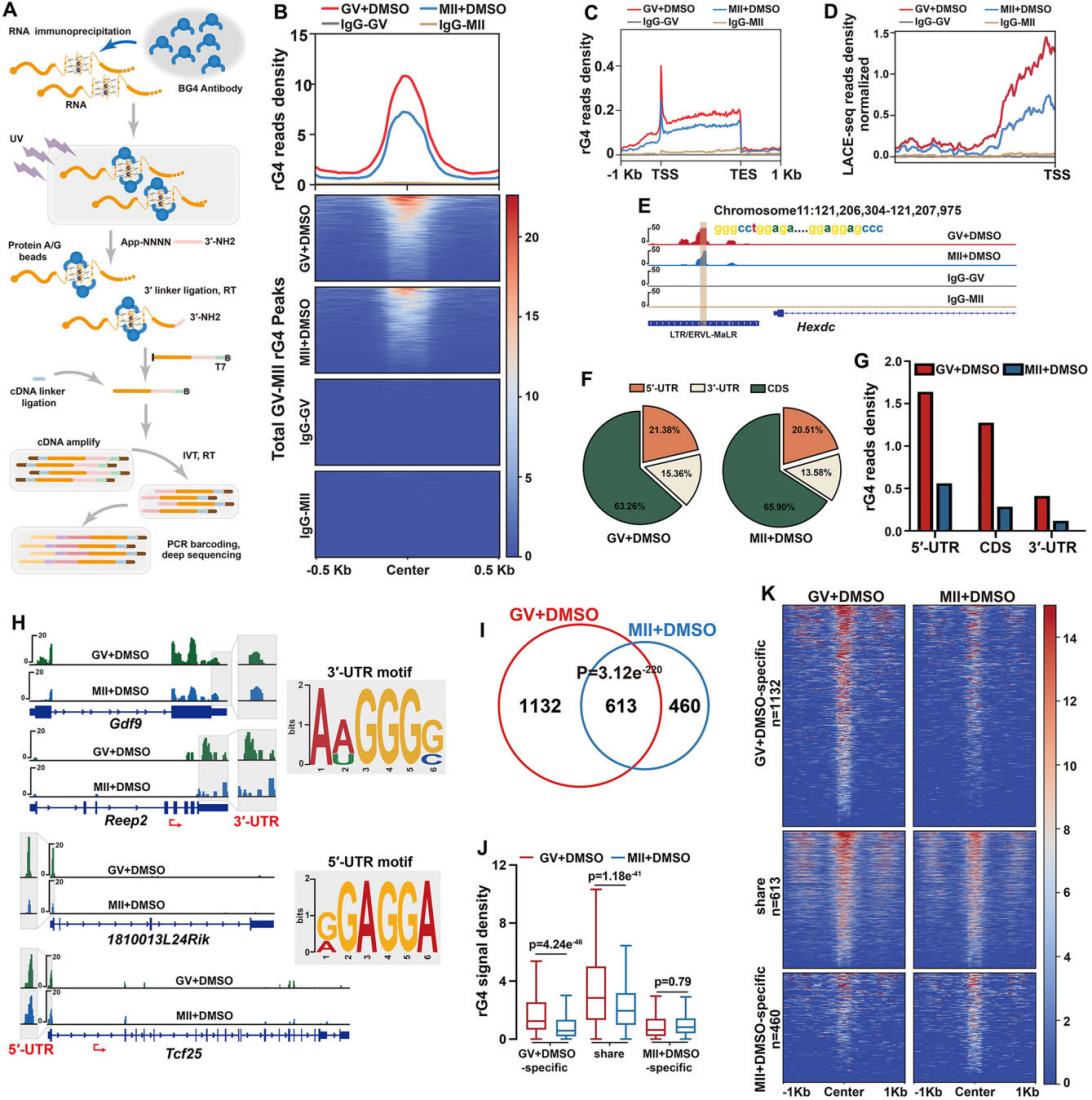
Changes of rG4 distribution in mouse oocytes during meiotic maturation. (**A**) Schematic of the improved rG4 detection method based on LACE-seq. Circled B, biotin modification. N, random nucleotide. (**B**) Read density and heatmap of rG4s in GV and MII oocytes across the ± 0.5 kb window around the rG4 peaks. IgG as a negative control. (**C**) Genomic metaplots showing relative signal intensity comparison of rG4s across the ± 1 kb window in GV and MII oocytes. TSS, transcription start sites; TES, transcription termination sites. (**D**) Genomic metaplots showing a relative signal intensity comparison of rG4s within the transposable elements before the TSS in GV and MII oocytes. (**E**) Snapshot of the rG4 peaks in LTR/ERVL-MaLR located upstream of the TSS of *Hexdc* in oocytes at the GV and MII stages. (**F**) Pie charts showing the distribution of rG4 peaks in GV and MII oocytes. (**G**) rG4 density calculated by RPKM in different regions of mRNA in GV and MII oocytes. (**H**) Snapshots of the rG4 peaks in representative genes. The inset shows the rG4s on 5′-UTRs and 3′-UTRs. (**I**) Venn diagram showing the overlap of the rG4-containing transcripts in GV and MII oocytes. They are divided into three clusters: GV-specific (1132), MII-specific (460), and shared by GV and MII oocytes (613). *P*= 3.12e^−220^ by two-tailed Student's t-test. (**J**) Box plot showing the rG4 signal densities of transcripts in three clusters shown in (**I**). The box indicates upper and lower quantiles, the thick line in the box indicates the median. *P* = 4.24e^−46^, *P* = 1.18e^−41^, and *P* = 0.79 by two-tailed Student's t-test. (**K**) Heatmap showing the rG4 signals across the ± 1 kb window around rG4 peaks in transcripts of three clusters. The numbers of transcripts are indicated (n).

G4-LACE-seq was performed according to published procedure [45] (Fig. 1A). Briefly, 50 oocytes were gathered in 1.5 ml Eppendorf tubes, added with 50 μl wash buffer, and lysed on ice for 10 min. Subsequently, 1 μl SUPERase (Ambion, AM2696) and 4 μl RQ1 DNase (Promega, M6101) were added to the lysate and incubated at 37°C for 3 min. The tubes were then incubated with 2 μg BG4 antibody and incubated at 4°C for 1 h. The samples on ice were then exposed to UV-C light twice at 400 mJ, after which 10 μl protein A/G beads were added to the samples and rotated at 4°C for 2 h. Following thorough washing steps, the RNAs captured by immunoprecipitation were fragmented, subjected to 3′-dephosphorylation and linker ligation, and then underwent reverse transcription on beads. Subsequently, first-strand complementaryDNA (cDNAs) were generated from protein A/G beads and captured by streptavidin C1 beads for pre-polymerase chain reaction (PCR), with the addition of a 3′-cDNA linker to synthesize double-stranded DNA, serving as the template for *in vitro* transcription (IVT). The IVT products were cleaned by eliminating the DNA template with Turbo DNase and further purified using Agencourt RNA Clean beads, according to the manufacturer's instructions. Thereafter, the linearly amplified RNA was converted into cDNA and then amplified by PCR using P7 and barcoded P5 index primers. The final PCR products, ranging in size from 250 to 500 bp, were extracted from a 2% agarose gel and purified using a gel purification kit (Qiagen, Catalog # 28 604) following the manufacturer's guidelines.

### LACE-seq data analysis

The adapter sequences of the raw reads were first removed using Cutadapt (v.1.14) [46] with the following parameters: -a AGATCGGAAGAGC-e 0.2 -m 12 -B AGATCGGAAGAGC –trim-n, and fastp (v. 0.12.4) [47] with the following parameters: -f 10 -F 10 -t 4 -T 4-overlap_len_require 10-overlap_diff_percent_limit 10. Clean reads were first aligned to the mouse pre-rRNA using the Bowtie software (v.2.3.5.1) [48], and the remaining unmapped reads were then aligned to the mouse (mm10) reference genome using hisat2 (v.2.2.1) [49] with default parameters. After removing the PCR duplicates using Sambamba (v.0.7.1) [50] with default parameters, deep Tools (v.3.5.1) [51] were used to generate bigwig files for visualization with the following parameters: bamcoverage, bs 10. We used the macs (v.2.2.7.1) [52] software to identify the peaks in all of the samples. The parameters used were as follows: keep-dup all-fe-cutoff, 2–p 0.05 –no model. And for motif analysis, LACE-seq peaks were first extended 30 nt to 5′ upstream, and obtained its corresponding sequence on the mm10 reference genome by bedtools (v.2.30.0) [53], then with default parameters of meme (v.4.11.2) [54] software to obtain their motif logo. A summary of the LACE-seq data generated in this study is listed in Supplementary Table S1.

### Immunofluorescence and confocal microscopy

Oocytes were fixed with 4% paraformaldehyde in phosphate-buffered saline (PBS) for 30 min at 25°C. The cells were permeabilized with 0.3% Triton X-100 in PBS for 25 min. Next, oocytes were transferred to 1% BSA in PBS for 1 h, subsequently incubated with primary antibody in blocking buffer for 1 h at 25°C. After washing in PBS, oocytes were incubated in secondary antibody for 30 min and 4′,6-diamidino-2-phenylindole (DAPI, 5 μg/ml) for 10 min at 25°C. The oocytes were washed thrice, transferred to SlowFade Gold Antifade Reagent (Life Technologies), and mounted on glass slides. The antibodies used in the experiments are listed in Supplementary Table S2. Imaging was performed using a Zeiss LSM710 confocal microscope. Semi-quantitative analysis of the fluorescence signals was conducted using ImageJ software.

### Circular dichroism and melting point assay

CD spectra were acquired using a Chirascan spectrophotometer (Applied Photophysics). RNAs were pre-annealed by heating at 95°C for 10 min in Tris–HCl buffer (10 mM, pH 7.4) with 100 mM KCl and cooled to 25°C. A quartz cuvette with a length of 1 mm was used to record the spectrum in the wavelength range of 220–340 nm with a 1 nm bandwidth, 1 nm step, and 0.5 s per point. Each curve was scanned three times. After setting the parameters, 300 μl of RNAs with a concentration of 5 μM was placed in a colorimetric dish. First, the CD spectra of RNA were collected. Then, the CD spectra of the RNA solutions mixed with different concentrations of BYBX (5, 10, 15, and 20 μM) were collected.

CD melting point assays were performed at 262 nm using a Chirascan spectrophotometer. Experiments were performed using 10 μM BYBX and 5 μM RNA in Tris–HCl buffer (10 mM, pH 7.4) containing 20 mM KCl. The temperature was gradually increased from 25 to 95°C, with a 1 nm step size, and 5 s per point. The collected data were normalized using Origin software.

### Fluorescence titration

The fluorescence spectra were recorded using an LS-45 fluorescence spectrometer (Perkin Elmer). The slit width of the colorimetric dish was 1 mm, and the optical diameter was 10 mm. The BYBX emission was acquired by exciting the sample in solution at 447 nm. The emission spectrum collection range of BYBX was 480–700 nm. All RNAs were pre-annealed by heating at 95°C for 10 min in Tris–HCl buffer (10 mM, pH 7.4) with 100 mM KCl. In the titration, RNAs were added into the solution containing 1 μM BYBX, and the final concentration of RNAs was varied from 0 to 1.9 μM. The mixture was stirred for 1 min until equilibrium was reached. The emission of the mixture was measured using a fluorescence spectrometer.

### Isothermal titration calorimetry assay

Isothermal titration calorimetry (ITC) experiments were conducted using a MicroCal PEAQ-ITC microcalorimeter (Malvern, USA). Oligonucleotides were initially annealed in a 25 mM KH_2_PO_4_, 60 mM KCl buffer (pH 7.4, containing 0.4% (v/v) DMSO) by heating them to 95°C in a water bath for 10 min. The solution was then cooled to 25°C and placed at 4°C overnight. The pre-annealed oligonucleotides (10 μM) in buffer were placed in the sample cell, while 40 μL of BYBX (100 μM) in the same buffer was loaded into the syringe. BYBX was mixed with the sample by stirring the syringe at 750 rpm at 25°C. Nineteen injections were made, each lasting 4 s with an interval of 150 s. For the blank titration, the ligand was injected into the cells containing only the buffer solution. The heat generated by the interaction was determined by subtracting the blank heat from the heat of the ligand-nucleic acid titration. Finally, the corrected data were fitted into the built-in binding model to obtain the binding enthalpy. The binding affinity (K_D_) was calculated by applying the built-in curve fitting model using ‘One Set of Sites’ (MicroCal PEAQ-ITC, Malvern, USA).

### Detection of RNA-binding protein in 293T cells

RBPs were detected according to a published protocol [55]. 293T cells were cultured in DMEM containing 10% fetal bovine serum for 24 h, washed with PBS, and irradiated twice with UV-C light on ice at 400 mJ. Next, the cells were lysed in PEB buffer (20 mM Tris–HCl pH 7.4, 100 mM KCl, 5 mM MgCl_2_, 0.5% NP40), and rocked for 20 min at 4°C. The protein samples were incubated with 1 μg/μL biotinylated oligo (dT), 2X TENT (20 mM Tris–HCl pH 8.0, 2 mM EDTA, 500 mM NaCl, 1% Triton X-100), 200X PMSF, 1000X Aproptin, 400 μM VRC, and 100 U/μl RRI. The biotinylated oligo (dT) sequence used in this study is as follows: 5′-biotin-ACCGT*-GGCCATTTTTTTTTTTTTTTTTTTT-3′; *: The 5th T contained a biotin modification. Incubation was performed for 30 min with rocking at 25°C. Each assembly added streptavidin-beads and was then rocked for 2 h at 4°C. The beads were adsorbed onto a magnet and washed thrice with 1X TENT. The samples were added to 1X sodium dodecyl sulphate (SDS) and denatured for 10 min at 95°C. Samples were separated using sodium dodecyl sulfate-polyacrylamide gel electrophoresis. The gel samples were cut and subjected to mass spectrometric analysis.

Proteins with an input group >0 were retained after annotating the Liquid Chromatography-Tandem Mass Spectrometry (LC-MS/MS) results using MaxQuant [56] software for the RBPs data analysis. Differentially expressed RBPs were classified based on tehe ratio of BYBX to Mock RBPs in the immunoprecipitation (IP) and input groups. Metascape [57] was used to perform functional enrichment analysis of the differentially expressed RBPs.

### Ribo-lite library sequencing and analysis

Ribo-lite experiments were performed using 500 oocytes, following a published protocol [58]. Oocytes were lysed in lysis buffer (20 mM Tris–HCl pH 7.5, 150 mM NaCl, 5 mM MgCl_2_, 1 mM DTT, 100 μg/ml CHX, 1% Triton X-100, 25 U/μl Turbo DNase), treated with RNase Inhibitor, and reactions were halted with SUPERase (Ambion, AM2696) after rotating for 45 min. Ribosomes were pelleted through centrifugation over a sucrose cushion (1 M sucrose in 20 mM Tris–HCl PH 7.5, 150 mM NaCl, 5 mM MgCl2, 1 mM DTT, 100 μg/ml CHX, and 20 U/ml SUPERase) for 4 h at 7600 rpm, resuspended in pellet buffer (10 mM Tris pH 7.5 and 1% SDS), and clarified with TRIzol and chloroform. Then, isopropanol and glycogen were added to the supernatant and frozen at −20°C overnight. Glycogen was centrifuged for 40 min at 12 000 × g, washed with 1 mL of 80% ethanol, and then resuspended in nuclease-free water. The samples and markers were respectively mixed with Gel Loading Buffer II (Thermo Fisher, AM8546G), and denatured for 90 s at 80°C, then separated on a 15% (wt/vol) polyacrylamide TBE-urea gel. RNA was extracted by adding RNA extraction buffer (300 mM sodium acetate pH 5.5, 1 mM EDTA and 0.25% SDS), snap-frozen at −80°C for 30 min, and thawed at 25°C overnight. The extract was then briefly centrifuged to collect the liquid at the bottom. The RNA was precipitated using isopropanol and glycogen. Libraries were prepared using the D-Plex Small RNA-seq Kit (Diagenode, C05030001) and sequenced on Illumina platforms with 150 bp paired-end reads. The analyses of Ribolite data were consistent with a published article, but using the mouse (mm10) reference genome [58]. The marker sequences used in this study were: NI-800:5′-AUGUACACUAGGGAUAACAGGGUAAUCAACGCGA/3Phos/; NI-801:5′-AUGUUAGGGAUAACAGGGUAAUGCGA/3Phos/. A summary of the ribo-lite data generated in this study is listed in Supplementary Table S3.

### RNA-seq library preparation and data analysis

The oocytes were collected at the indicated time points after treatment with DMSO or BYBX (10 oocytes per sample). Each sample was lysed using 4 μl lysis buffer (0.2% Triton X-100, RNase inhibitor, dNTPs, oligo-dT primers, and 100 pg external RNA controls consortium (ERCC) mRNA spike-in) and immediately used for cDNA synthesis based on the Smart-seq2 method. Sequencing libraries were constructed from 500 pg of amplified cDNA using a TruePrep DNA Library Prep Kit V2 for Illumina (Vazyme, TD503) according to the manufacturer's instructions. Barcoded libraries were pooled and sequenced on the Illumina HiSeq X Ten platform using 150 bp paired-end reads.

For RNA-seq data analysis, adapters were removed from the raw data by trim-galore (v.0.6.7) and aligned to the mouse (mm10) reference genome using hisat2 (v.2.2.1) [59] with the default parameters. Uniquely mapped reads were assembled into transcripts guided by reference annotation (University of California at Santa Cruz [UCSC] Gene Models) using Cufflinks (v2.2.1) [59]. The level of expression of each gene was quantified as the Fragments Per Kilobase of exon model per Million mapped fragments (FPKM) and normalized to the ERCC spike-in. The samples prepared in different batches were normalized to the GV-stage oocyte samples from each batch. Statistical analyses were performed using R software (http://www.rproject.org). Functional annotation was performed using the R package Cluster Profiler (version 4.0) [60]. A summary of the RNA-Seq data generated in this study is presented in Supplementary Table S4.

### In vitro transcription and microinjections

The expression vectors were linearized and subjected to phenol/chloroform extraction and ethanol precipitation. The linearized DNAs were *in vitro* transcribed using the SP6 mMACHINE^®^ Kit (Invitrogen, AM1340) followed by the Poly (A) Tailing Kit (Invitrogen, AM1350), according to the manufacturer's instructions. mCherry mRNA was transcribed using the T7 mMACHINE^®^ Kit (Invitrogen, Carlsbad, CA, USA; AM1344) and *in vitro* polyadenylated using a Poly (A) Tailing Kit. The mRNAs were recovered by lithium chloride precipitation, and resuspended in nuclease-free water at the concentration of 500 ng/μl.

The microinjections were performed using an Eppendorf TransferMan NK2 micromanipulator. For microinjection, fully grown GV oocytes were incubated in M2 medium with 2 μM milrinone. Denuded oocytes were injected with 5–10 pl mRNA per oocyte into the cytoplasm. After injection, oocytes were washed and then cultured in M16 medium at 37°C with 5% CO_2_.

### Western blot

The oocytes were collected in 1X SDS loading buffer and denatured for 15 min at 95°C. Total oocyte protein lysates were separated by sodium dodecyl sulfate-polyacrylamide gel electrophoresis and transferred to PolyVinylideneFluoride (PVDF) membranes under constant current via semi-dry transfer. The PVDF membranes were washed with 0.1% Tween-20 in Tris-buffered saline (TBST) and blocked in TBST buffer containing 5% non-fat milk for 40 min at 25°C. Next, the PVDF membranes were incubated with the primary antibody in blocking buffer at 4°C overnight and washed three times with TBST for 15 min. The target proteins were then incubated in horseradish peroxidase (HRP)-linked secondary antibody for 45 min at 25°C and washed three times in TBST. Bound signals were detected using Super Signal West Femto Maximum Sensitivity Substrate (Cyanagen). The antibodies used in the experiments are listed in Supplementary Table S2. The mean gray scale signal was measured and quantified using ImageJ software.

### Detection of protein synthesis in the oocytes

The oocytes were cultured in M16 medium supplemented with 50 μM homopropargylglycine (HPG) from Click-iT^®^ HPG Alexa Fluor Protein Synthesis Assay Kit (Thermo Fisher Scientific) for 1 h. After washing with PBS, the oocytes were fixed in 4% formaldehyde for 30 min at 25°C, then permeabilized and washed as the procedures described in immunofluorescence and confocal microscopy. Alexa Fluor 488 was conjugated to the nascent protein using a Click-iT^®^ cell reaction kit for 30 min and washed with rinse buffer. DNA was stained with DAPI and mounted on slides as aforementioned. The mean fluorescence signal was measured and quantified using ImageJ software.

### Statistical analysis

Results are presented as means ± SEM. Most of the experiments included at least three independent samples and were repeated at least thrice. The results of the two experimental groups were compared using two-tailed unpaired Student's t-tests. Statistically significant values of *P* < 0.05, *P* < 0.01, and *P* < 0.001 determined by two-tailed Student's t-tests are indicated by asterisks (*), (**), and (***), respectively. ‘n.s.’ indicates non-significant.

## Results

### Distribution of rG4 in mouse oocytes during meiotic maturation

As the oocytes are low-input cells, we used BG4-LACE-seq to detect rG4 in 50 mouse oocytes. BG4-LACE-seq adjusts the experimental step of UV-cross-linking and antibody incubation based on LACE-seq and depends on rG4-mediated reverse transcription termination on the BG4 antibody-rG4 complex and the subsequent linear amplification of terminating cDNA ends (Fig. 1A). The two replicates showed a high correlation (Supplementary Fig. S1A). The heatmap shows that rG4 density was enriched by the BG4 antibody when compared to that by immunoglobulin G (IgG) in 50 oocytes (Fig. 1B). Total rG4 density was higher at the GV stage than at the MII stage (Fig. 1B). The genomic distribution of rG4 was similar in the GV and MII oocytes, with enriched rG4 signals observed in the transcription start sites (TSS) (Fig. 1C). Interestingly, significant signals were detectable in the region before the TSS compared to the IgG group. Most of the signals in this region were concentrated on transposable elements (Fig. 1D). Fig. 1E demonstrates that the LTR/ERVL-MaLR elements, located upstream of the TSS of *Hexdc*, were rich in rG4 signals, compared to the IgG group.

In the mRNAs, the coding sequences (CDSs) contained more rG4 peaks than the 5′-UTRs or 3′-UTRs (Fig. 1F). However, normalizing by the length of each region, Reads Per Kilobase per Million mapped reads (RPKM) indicating rG4 density in 5′-UTRs was higher than that in CDSs and 3′-UTRs. In addition, the rG4 density in GV oocytes was higher than that in MII oocytes (Fig. 1G), as shown in Fig. 1B. Consensus G-rich motifs were identified by analyzing LACE-seq results in GV and MII oocytes (Supplementary Fig. S1B). Classical G-rich motifs were detected in the UTRs of the oocyte transcripts (Fig. 1H). A snapshot of representative transcripts also showed that rG4 signals decreased during meiotic maturation (GV–MII transition) (Fig. 1H).

In GV and MII oocytes, 1745 and 1073 rG4-containing transcripts were identified, respectively. Around 57% of rG4-containing transcripts in MII oocytes also contained rG4s in GV oocytes. (*P*= 3.12e^−220^, Fig. 1G). The transcripts were divided into three groups: Group 1 contained 1132 transcripts with rG4s only at the GV stage (GV-specific rG4s); Group 2 contained 613 transcripts with rG4s at both the GV and MII stages (shared rG4s); and Group 3 contained 460 transcripts with rG4s only at the MII stage (MII-specific rG4s). Even among the transcripts (Group 2) with shared rG4s, rG4 density was significantly higher at the GV stage than at the MII stage (Fig. 1J and K), whereas rG4 density was low among transcripts containing MII-specific rG4s. (Fig. 1J). These data indicate that rG4 density was high in GV oocytes but dramatically decreased at the MII stage.

### BYBX stabilizes rG4s in mouse oocytes and disrupts oocyte meiotic maturation

**Figure 2.**
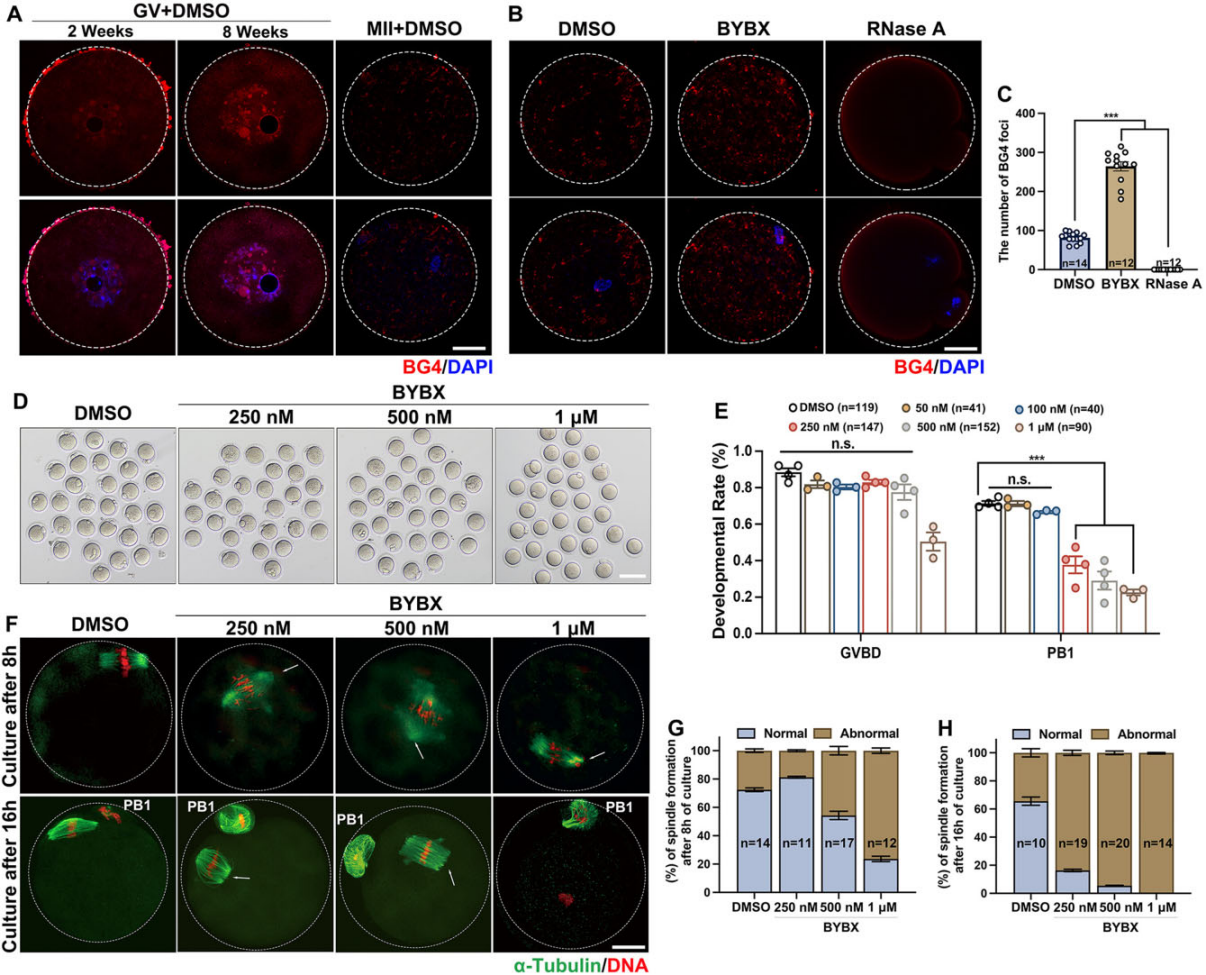
Impact of BYBX on mouse oocyte meiotic maturation. (**A**) Confocal microscopic images of DAPI (blue) and BG4 (red) immunofluorescence in mouse oocytes. GV, germinal vesicle; MII, metaphase II. Scale bar = 20 μm. (**B**) DAPI (blue) and BG4 (red) immunofluorescence in MII oocytes after BYBX and RNase A treatment. Scale bar = 20 μm. (**C**) The number of BG4 foci in the MII oocytes after BYBX treatment. (**D**) Representative images of the oocytes cultured 16 h after BYBX treatment. Fully grown GV oocytes were collected from PMSG-primed (44 h) mice. Scale bar = 100 μm. (**E**) Rates of GVBD and polar body-1 (PB1) emission in oocytes cultured without or with BYBX treatment. (**F**) Confocal microscopy results showing spindle assembly in the oocytes cultured for 8 and 16 h after BYBX treatment. Scale bar = 20 μm. (**G**-**H**) Percentage (%) of normal spindle in oocytes after 8 hours (**G**) and 16 h (**H**) of culture in medium containing DMSO or BYBX.

To understand the impact of rG4s in oocyte meiosis, we experimentally stabilized rG4s using the fluorescent rG4-ligand BYBX. Immunofluorescence staining using a G4-specific BG4 monoclonal antibody showed that rG4s were distributed in the nucleus and cytoplasm of growing oocytes obtained from 2-week-old mice. As the oocytes grew, the rG4 signals were found to be increased in fully grown oocytes collected from 8-week-old mice. rG4 presented as speckles in the GVs but became more diffusely distributed in the ooplasm of MII oocytes (Fig. 2A). We observed a significant increase in cytosolic BG4 foci in MII oocytes after BYBX treatment compared to control oocytes, whereas almost no BG4 foci were detected in the cytoplasm after RNase A treatment (Fig. 2B and C), indicating that BYBX stabilized rG4s in oocytes, as expected. We also cultured oocytes with another rG4-stabilizing probe, P67 [61], and performed immunofluorescence staining using the BG4 antibody. The number of BG4 foci in the cytoplasm of MII oocytes treated with P67 was also significantly increased compared with the control group (Supplementary Fig. S2C and D).

In fully grown GV oocytes cultured with BYBX, meiotic resumption, characterized by GVBD, was not affected, but polar body 1 (PB1) emission rates decreased with increasing BYBX concentration (Fig. 2D and E). Proper spindle assembly is critical in oocyte meiosis. Although a small proportion of BYBX-treated oocytes released PB1 and developed to the MII stage (Fig. 2D), they contained abnormal spindles, and chromosomes were not aligned at the equatorial plates at both the metaphase I (MI) (8 h in culture) and MII (16 h in culture) stages (Fig. 2F–H; Supplementary Fig. S1C and D). We also cultured oocytes with another rG4-stabilizing probe, P67, and a dG4-stabilizing probe, CYTO-4C [62]. Consistent with the BYBX incubation results, the 1 μM P67 treatment significantly reduced the PB1 emission rate of oocytes (Supplementary Fig. S1G and H). However, after treatment with the dG4-stabilizing probe CYTO-4C, we found that the stabilization of dG4s did not affect oocyte maturation (Supplementary Fig. S1I and J). Similarly, abnormal spindles were observed in oocytes treated with P67 at both MI and MII stages (Supplementary Fig. S1K–M). In contrast, oocytes treated with CYTO-4C still formed normal spindles (Supplementary Fig. S1K–M). These results suggest that rG4s, but not dG4s, accumulation disrupts oocyte maturation.

### BYBX disrupts the dynamic turnover of rG4s by stabilizing them during oocyte meiotic maturation

**Figure 3.**
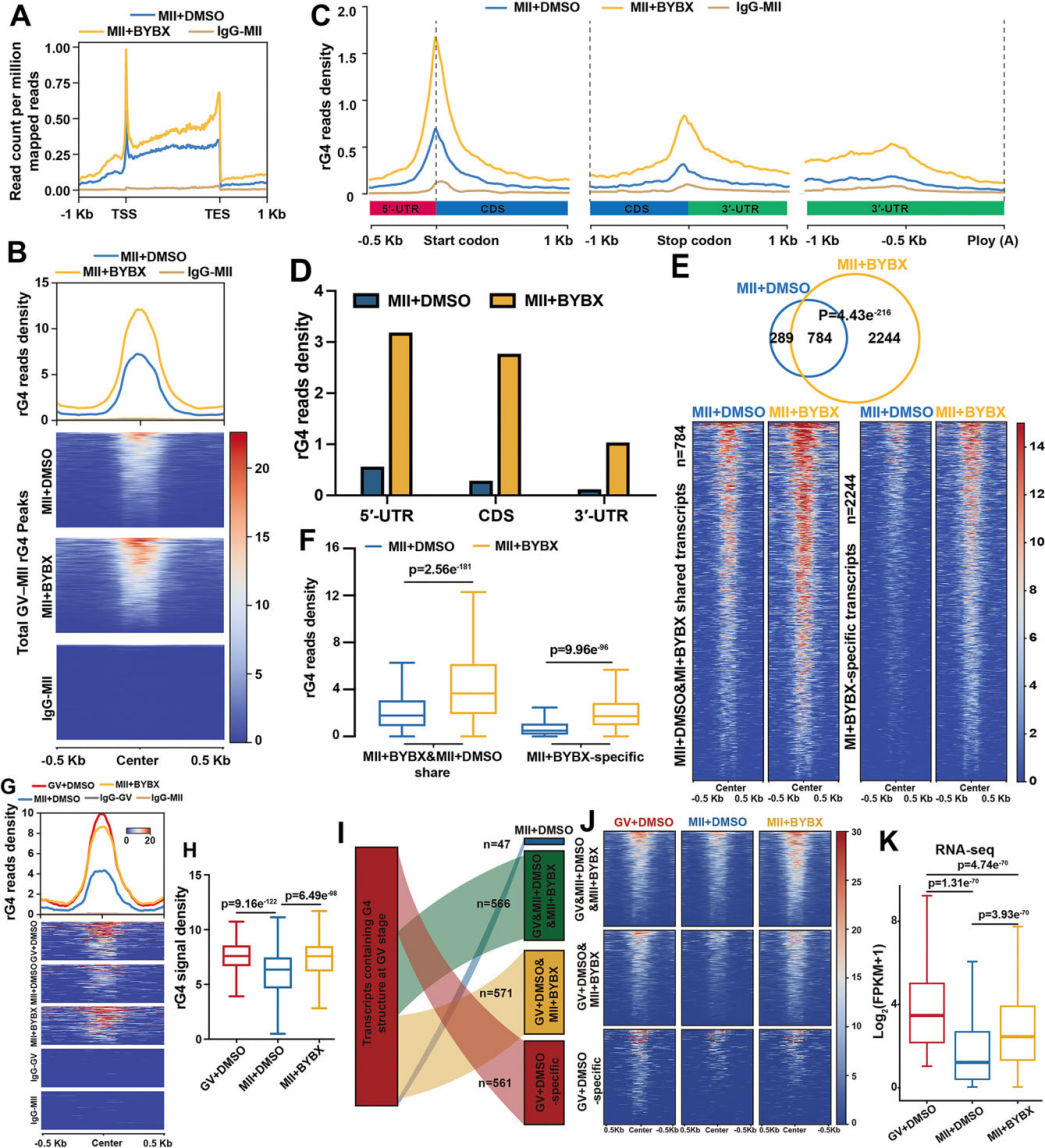
Changes of rG4 distribution in mouse oocytes after BYBX treatment during meiotic maturation. (**A**) Genomic metaplots showing relative signal intensity of rG4s across the ± 1 kb window in the MII oocytes with DMSO and treatment. (**B**) rG4 read density and heatmap across the ± 0.5 kb window around the rG4 peaks in MII oocytes. IgG as a negative control. (**C** and **D**) rG4 distributions (**C**) and densities (**D**) at different mRNA locations in MII oocytes with DMSO and BYBX treatment. (**E**) Venn diagram (above) showing the overlap of rG4-containing transcripts in MII oocytes with DMSO and BYBX treatment. Heatmap (bottom) showing the changes of the rG4 signals in GV and MII oocytes. *P*= 4.43e^−216^ by two-tailed Student's t-test. The number of analyzed transcripts is indicated (n). (**F**) Box plot showing ratio of rG4 signal density in the MII oocytes with DMSO and BYBX treatment. The box indicates upper and lower quantiles, and the line in the box indicates the median. *P*= 2.56e^−181^ and *P* = 9.96e^−96^ by two-tailed Student's t-test. (**G**) rG4 read densities and heatmaps of rG4s across the ±0.5 kb window around rG4 peaks in transcripts containing downregulated rG4s during normal GV–MII transition. (**H**) Box plots showing the rG4 signal densities in (**G**). The box indicates upper and lower quantiles, the thick line in the box indicates the median. *P* value by two-tailed Student's t-test. (**I** and **J**) Alluvial plots (**I**) and heatmaps (**J**) showing rG4 signal density changes in oocytes after BYBX treatment. Transcripts containing rG4s at the GV stage were selectively analyzed. K: Box plot showing transcript expression levels of GV oocytes and MII oocytes with DMSO and BYBX treatment.

We performed BG4-LACE-seq on MII oocytes treated with BYBX (1 μM). rG4 density increased after BYBX treatment, especially in the TSS and transcription termination site regions (Fig. 3A). The heatmap shows that the total rG4 signals identified by BG4-LACE-seq increased in MII oocytes after BYBX treatment compared with those in oocytes treated with DMSO as a control (Fig. 3B). Quantified analysis of the G4 distribution in the mRNAs showed G4 density had a similar raised multiplier in start codon, stop codon, and 3′-UTR (Fig. 3C and D). These results show that BYBX stabilizes rG4 without regional preferences. Among the rG4-containing transcripts detected in BYBX-treated oocytes, 784 were rG4-bearing in control oocytes, while 2244 transcripts were rG4-negative in control oocytes (Fig. 3E). Heatmap (Fig. 3E) and further quantification results (Fig. 3F) showed that even in the rG4-positive transcripts shared by control and BYBX-treated oocytes, rG4 levels were higher in BYBX-treated oocytes than those in DMSO-treated oocytes, demonstrating the significant accumulation of rG4s in BYBX-treated oocytes.

Furthermore, BYBX treatment prevented rG4 clearance during the GV–MII transition (Fig. 3G and H). Only in 561 transcripts, meiotic maturation-coupled rG4 removal was not affected by BYBX treatment; in 571 and 566 transcripts, rG4 removal was completely or partially blocked, respectively, after BYBX treatment (Fig. 3I and J). We also further analyzed the overall transcript levels. Consistent with previous studies [6, 8], the GV-MII transition is accompanied by significant degradation of maternal mRNA. However, the transcript levels in BYBX-treated MII oocytes were not decreased and were higher than those in the control group, indicating that BYBX treatment does not affect RNA stability (Fig. 3K). Gene ontology (GO) and network analyses revealed that rG4s stabilized by BYBX were preferentially found in transcripts involved in mRNA processing and translational regulation (Supplementary Fig. S2A). Collectively, these results have confirmed at the transcriptome level that BYBX disrupts the dynamic turnover of rG4s by stabilizing them during oocyte maturation.

### BYBX interacts with and stabilizes the rG4 motif in the *Zar1* 5′-UTR

**Figure 4.**
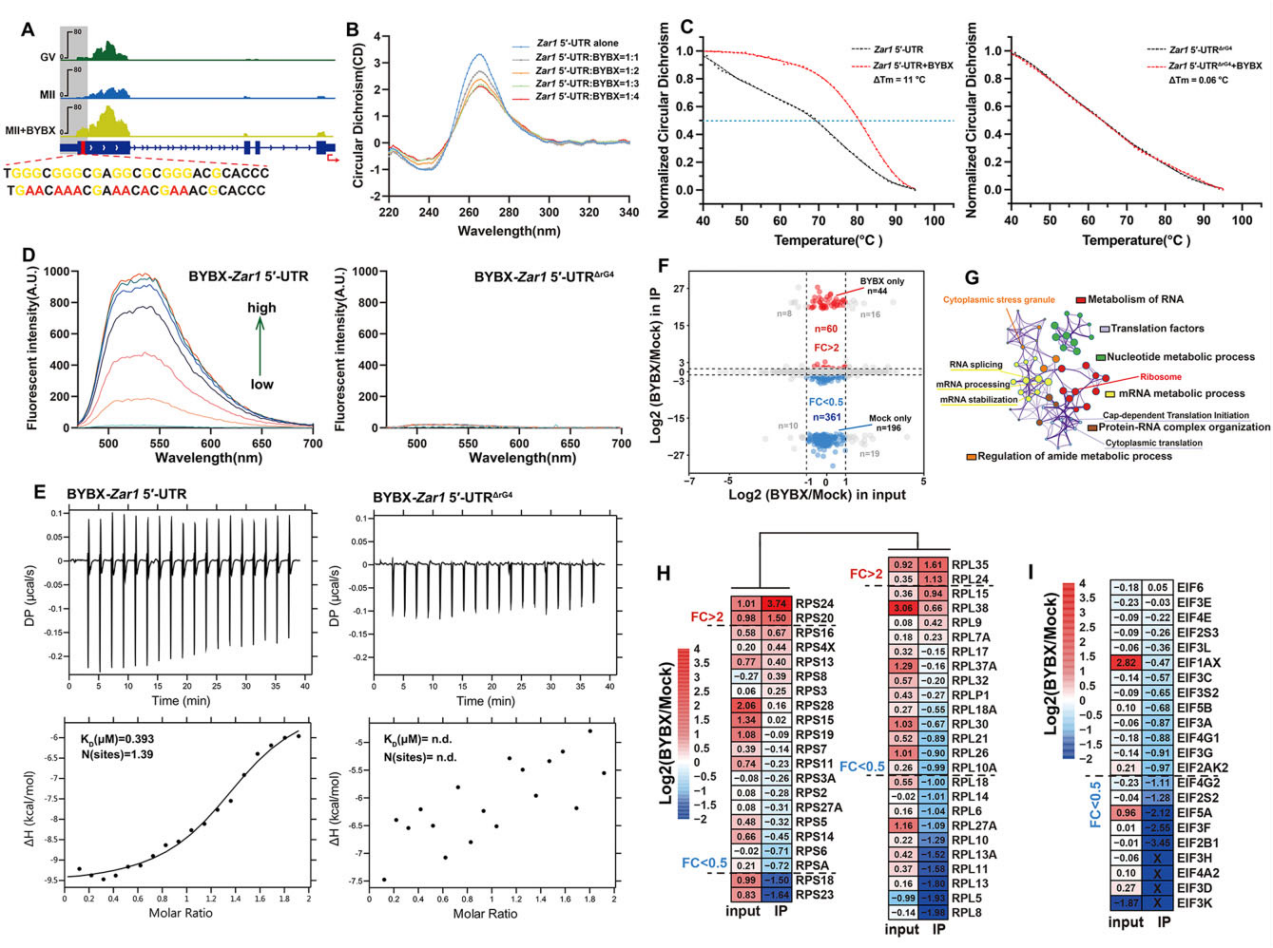
Impacts of BYBX on rG4s in the 5′-UTRs and the interactions between mRNAs and RBPs. (**A**) Snapshots showing rG4s in *Zar1* in GV and MII oocytes. Sequences of rG4 and mutated sequences (ΔrG4) in *Zar1* 5′-UTR are shown at the bottom. (**B**) CD spectra of BYBX with *Zar1* 5′-UTR in a Tris–HCl buffer solution (10 mM, pH 7.4, containing 100 mM KCl) at 25°C. The concentration of the oligonucleotides used was 5 μM. (**C**) Normalized CD signal of *Zar1* 5′-UTR and *Zar1* 5′-UTR^ΔrG4^ during the melting process. A magnified view of the CD signal from 20°C to 40°C is shown at the upper right. The concentrations of BYBX and RNAs were 10 and 5 μmol, respectively. (**D**) Fluorescence titration spectra of BYBX with *Zar1* 5′-UTR. The titrations were conducted in a Tris–HCl buffer (10 mM, pH 7.4) containing 100 mM KCl. Fluorescence measurements were performed at 25°C. The concentration of BYBX was 1 μM. The arrow indicates the concentration of RNAs ranged from 0 to 1.9 μM in the titration series. (**E**) ITC for the interaction of BYBX with *Zar1* 5′-UTR and *Zar1* 5′-UTR^ΔrG4^. The isothermal plots of BYBX were in the presence of different nucleic acids in 25 mM KH_2_PO_4_, 60 mM KCl buffer (pH 7.4). K_D_ (μM) indicates the dissociation constant of BYBX with oligonucleotides determined with ITC at 25°C. N represents the stoichiometry of Guest–Host interaction obtained in ITC experiments. n.d. denotes not determined because the ligand-oligonucleotide binding signal was too weak for estimation. (**F**) Scatter plot showing the BYBX-induced changes of RBP levels (*x*-axis) and the changes of RNA-bound RBPs (*y*-axis) detected by protein mass spectrometry. (**G**) Network analysis of the enriched GO terms of RBPs with downregulated RNA interactions after BYBX treatment. (**H**-**I**) Heat map showing BYBX-induced changes of ribosomal proteins (**H**) and eukaryotic translation initiation factors (**I**) before and after RNA immunoprecipitation detected by protein mass spectrometry.

ZAR1 is one of the best-known maternal factors involved in mRNA processing and translational regulation. As shown in the snapshot, the G4 signals in *Zar1* 5′-UTR significantly increased after BYBX treatment (Fig. 4A). To further evaluate the specificity of BG4-LACE-seq, and the contribution of BYBX to rG4 stabilization, we synthesized the *Zar1* 5′-UTR containing rG4 or with rG4 disruption (ΔrG4). *Zar1* 5′-UTR forms a parallel G-quadruplex conformation under the condition with K^+^ ions (Fig 4B). With the increase of BYBX concentration, the CD absorption of *Zar1* 5′-UTR at 262 nm was affected (Fig. 4B). The results confirmed that rG4 is formed in the *Zar1* 5′-UTR, and BYBX interacts with this rG4 structure.

Melting curves are commonly used to evaluate the relative binding strengths of G4-bounding ligands. The G4 structure was found to be stable at low temperatures but began to dissociate as the temperature increased, causing the CD signals to change. When the melting temperature (Tm) was reached, the G4 structure changed and the CD signal dropped sharply. The melting curve trend of *Zar1* 5′-UTR is consistent with the previous melting curve of rG4s, and the markedly increased melting points of the complex (ΔTm = 11°C), further confirming that the rG4 structure was stabilized upon *Zar1* 5′-UTR interacting with BYBX (Fig. 4C). However, the melting curve of *Zar1* 5′-UTR^ΔrG4^ does not show a transition trend, and the CD signal decreases at the same rate, indicating that there is no G4 structure in *Zar1* 5′-UTR^ΔrG4^. Meanwhile, *Zar1* 5′-UTR^ΔrG4^ was not able to produce a significantly increased ΔTm (ΔTm = 0.06°C) in the assay (Fig. 4C), indicating that the interaction of BYBX with *Zar1* 5′-UTR^ΔrG4^ was much weaker than that of *Zar1* 5′-UTR.

In addition, we used fluorescent titration to determine the binding ability of BYBX to *Zar1* 5′-UTR. *Zar1* 5′-UTR and *Zar1* 5′-UTR^ΔrG4^ were continuously added to the BYBX solution until the fluorescence intensity was saturated. The fluorescence signals (λex = 447 nm, λem = 538 nm) were enhanced 50-fold when BYBX was interacted with *Zar1* 5′-UTR compared to BYBX alone, while the *Zar1* 5′-UTR^ΔrG4^ exhibited almost no emission under the same assay conditions (Fig. 4D). To detect the interaction between the *Zar1* 5′-UTR and BYBX, we applied ITC to obtain the dissociation constant. The two interacting biomolecules are typically placed in separate syringe pumps, and a control system is used to mix the two sample solutions in a specific proportion. The released heat during this process is detected, enabling real-time monitoring and measurement of the interaction between the biomolecules. The binding affinity of BYBX toward the *Zar1* 5′-UTR was estimated by ITC assays (*K_D_* = 0.393 μM). In contrast, the *Zar1* 5′-UTR^ΔrG4^ substrates exhibited no binding affinity with the ligand (Fig. 4E). These results further demonstrate that BYBX strongly interacts with the *Zar1* 5′*-*UTR. Taken together, these *in vitro* results demonstrate the specificity of BG4-LACE-seq and confirm that BYBX is highly selective in targeting rG4s.

### The rG4s in the 5′-UTR disrupt translation initiation activity during oocyte maturation

rG4-induced conformational changes are likely to directly affect interactions between mRNAs and RBPs. Therefore, we employed an interactome capture technique to identify the RBPs whose RNA binding to GV oocytes was affected by BYBX treatment. Given the rarity and difficulty of obtaining oocytes, this experiment used 293T cells to investigate the changes in RBP binding to RNA following BYBX treatment. After incubation with BYBX, the binding of 361 and 60 RBPs to mRNAs was downregulated (FC[BYBX/DMSO] < 0.5) and upregulated (FC[BYBX/DMSO] > 2), respectively. Among these, the RNA binding of the 196 RBPs was completely blocked after BYBX treatment (Fig. 4F). GO analysis revealed that RBPs that failed to bind to RNAs after BYBX treatment were normally involved in the structural constituents of ribosomes, translation, mRNA processing, and cytoplasmic stress granules (Fig. 4G).

Typical translation initiation in eukaryotic cells begins with the assembly of the 43S pre-initiation complex. The 60S ribosomal subunit is recruited by eukaryotic translation initiation factor 5B (eIF5B) to produce an 80S initiation complex that is ready to begin protein synthesis [63]. BYBX treatment decreased the RNA-binding ability of the 60S ribosomal subunits; the binding of 10 ribosomal proteins of large subunits (RPL) to RNA decreased more than 2-fold after BYBX treatment. The binding of 40S ribosome subunits to RNA was not significantly affected; only two ribosomal proteins of small subunits (RPS) binding to RNA were up- and down-regulated by more than 2-fold after BYBX treatment (Fig. 4H). These observations suggested that the accumulated rG4s on 5′-UTRs hinder the movement of the pre-initiation complex and recruitment of the 60S ribosome subunits, thus disrupting the translation activities. Additionally, the RNA binding of all the eIFs was impaired after BYBX treatment (Fig. 4I), suggesting that the translation initiation complex could not be assembled properly after rG4 stabilization, thus inhibiting the translation of mRNAs.

### Oocyte translatome affected by BYBX-induced rG4 stabilization

**Figure 5.**
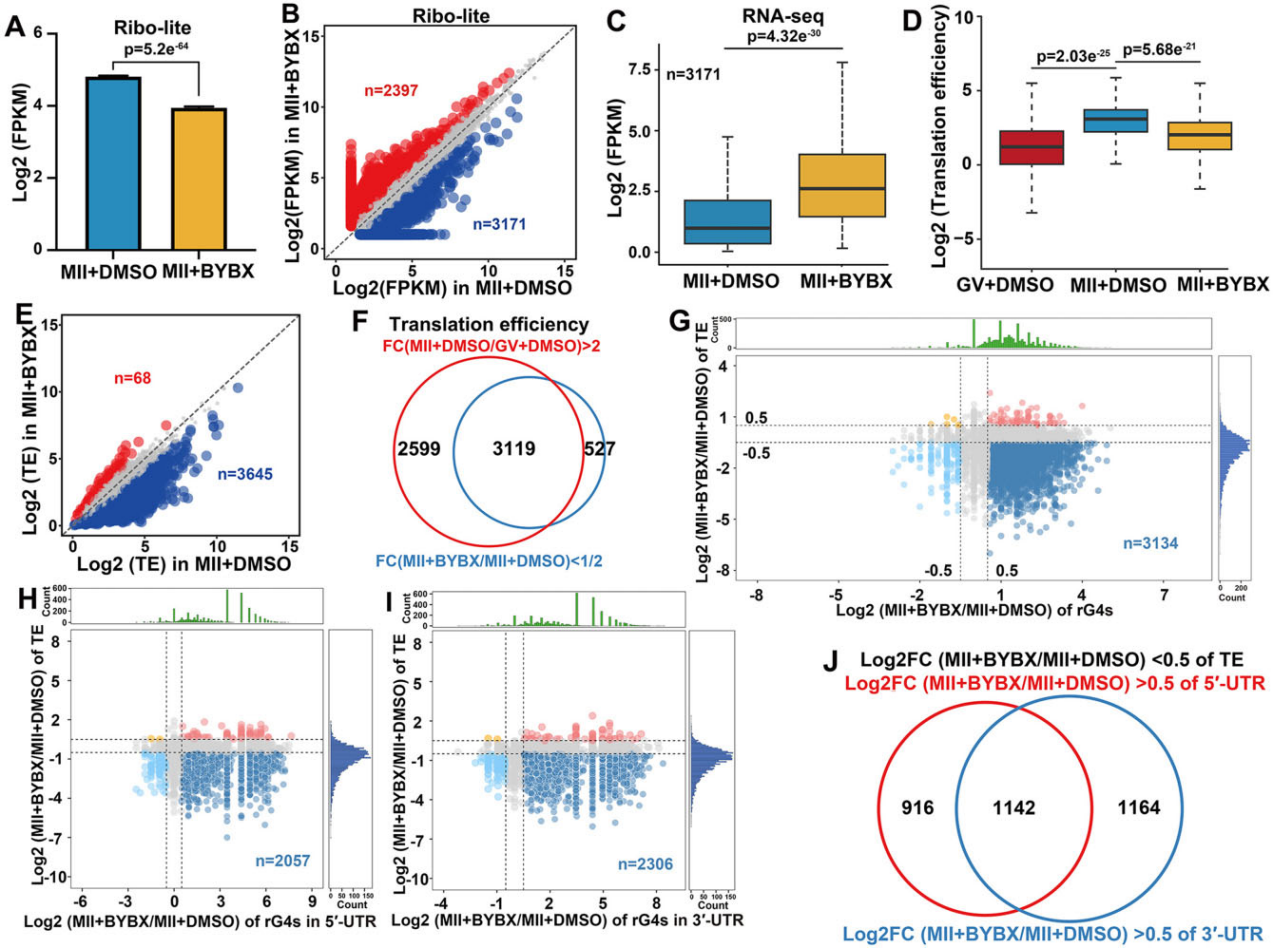
BYBX-induced rG4 stabilization affects mRNA translation efficiency during oocyte maturation. (**A**) Levels of ribosome-bound mRNAs were calculated through FPKM of Ribo-lite in MII oocytes with DMSO and 1 μM BYBX treatment. Error bars, SEM, *P*= 5.2e^−64^ by two tailed Student's t-test. (**B**) Scatter plot comparing the translatomes detected by Ribo-lite in MII oocytes with DMSO and 1 μM BYBX treatment. Translatomes decreased or increased more than 2-fold were highlighted with blue or red, respectively. (**C**) Transcripts with downregulated ribosome-binding after DMSO and 1 μM BYBX treatment in (**B**) were jointly analyzed with RNA-seq data. Box plot showing transcript expression levels of MII oocytes after BYBX treatment. The box indicates upper and lower quantiles, and the thick line in the box indicates the median. *P*= 4.32e^−30^ by two-tailed Student's t-test. (**D**) Box plot showing translational efficiencies (TEs) of transcripts in GV and MII oocytes. TE was calculated from the ratio of the RPFs (detected by Ribo-lite) to mRNA expression levels (detected by Smart-seq 2). The box indicates upper and lower quantiles, the thick line in the box indicates the median. *P*= 2.03e^−25^ and *P*= 5.68e^−21^ by two-tailed Student's t-test. (**E**) Scatter plot showing TE changes of transcripts in MII oocytes with DMSO and 1 μM BYBX treatment. Transcripts with TEs that decreased or increased more than 2-fold after BYBX treatment were highlighted with blue or red, respectively. (**F**) Venn diagrams showing the overlap between the transcripts with downregulated TEs in the MII oocytes after BYBX treatment and transcripts undergoing translational activation during the GV–MII transition. (**G**) Scatter plot showing the BYBX-induced change of rG4 levels in transcripts (*x*-axis) and TE changes of these transcripts (*y*-axis) in MII oocytes. (**H**-**I**) Scatter plot showing the BYBX-induced changes of rG4 levels in the 5′-UTRs (**H**) or 3′-UTRs of transcripts (**I**) (*x*-axis) and TE change of these transcripts (*y*-axis) in MII oocytes. (**J**) Venn diagrams showing the overlap between the transcripts containing rG4s in 5′-UTRs (dark blue in (**H**)) and in 3′-UTRs (dark blue in (**I**)) that exhibited downregulated TEs in the MII oocytes after BYBX treatment.

To further confirm that rG4s impair ribosome binding to mRNA and affect translation, we used low-input Ribo-seq (Ribo-lite) to detect ribosome-bound translatomes during the GV–MII transition after BYBX treatment. The two replicates showed a high correlation (Supplementary Fig. S2A). Ribosome-protected fragment (RPF) was decreased in MII oocytes after BYBX treatment (Fig. 5A). A total of 3171 and 2397 transcripts were found to have downregulated and upregulated changes in ribosome binding (2-fold), respectively (Fig. 5B). While 3171 transcripts had downregulated translational levels after BYBX treatment, their mRNA levels were upregulated in MII oocytes compared to controls, as detected by Smart-seq2 (Fig. 5C). Figs 3K and 5C excluded the influence of RNA stability after BYBX treatment. Because of the absence of de novo transcription and the existence of mRNA decay during the GV–MII transition, the results suggested that BYBX protected this subset of transcripts from degradation. This result also excludes the possibility that total mRNA reduction indirectly leads to translatome down-regulation.

To verify rG4s impaired oocyte translation, we used RPF/mRNA to indicate translational efficiency (TEs). During oocyte maturation, TEs were upregulated, but BYBX treatment significantly decreased TEs in MII oocytes (Fig. 5D). During oocyte maturation, TEs of 5718 transcripts were upregulated (FC[MII/GV] > 2). In contrast, only 163 transcripts showed downregulated TEs (FC[MII/GV] < 0.5) (Supplementary Fig. S2C). These results recapitulate previous findings that there is significant translation activation during oocyte maturation and confirm their reliability [7, 64, 65]. The Ribo-lite results showed that the TEs of almost all the transcripts in the BYBX-treated MII oocytes were downregulated (FC[MII/GV] < 0.5) (Fig. 5E). Among these, 85% (3119) were transcripts that underwent translational activation during oocyte maturation (Fig. 5F). Meanwhile, 3134 transcripts with decreased TEs showed rG4 accumulation (Fig. 5G). These results suggest that the accumulation of rG4 impairs translation in maturing oocytes.

As depicted in Fig. 1, we observed that G4 structures were predominantly enriched in the 5′-UTR, with a certain degree of enrichment also detected in the 3′-UTR. Both the 5′- and 3′-UTRs are crucial regions for regulating translation. After BYBX incubation, 2058 and 2306 transcripts with rG4s on the 5′- and 3′-UTRs exhibited decreased TEs, respectively (Fig. 5H and I). Among these, 55% (1 142) of transcripts with rG4s in the 5′-UTR also had rG4s in their 3′-UTR (Fig. 5J and Supplementary Fig. S2C), suggesting that the accumulation of rG4s on both the 5′- and 3′-UTRs might jointly impair translation activation in oocytes.

### rG4s in the 5′-UTR impair translation of maternal transcripts

**Figure 6.**
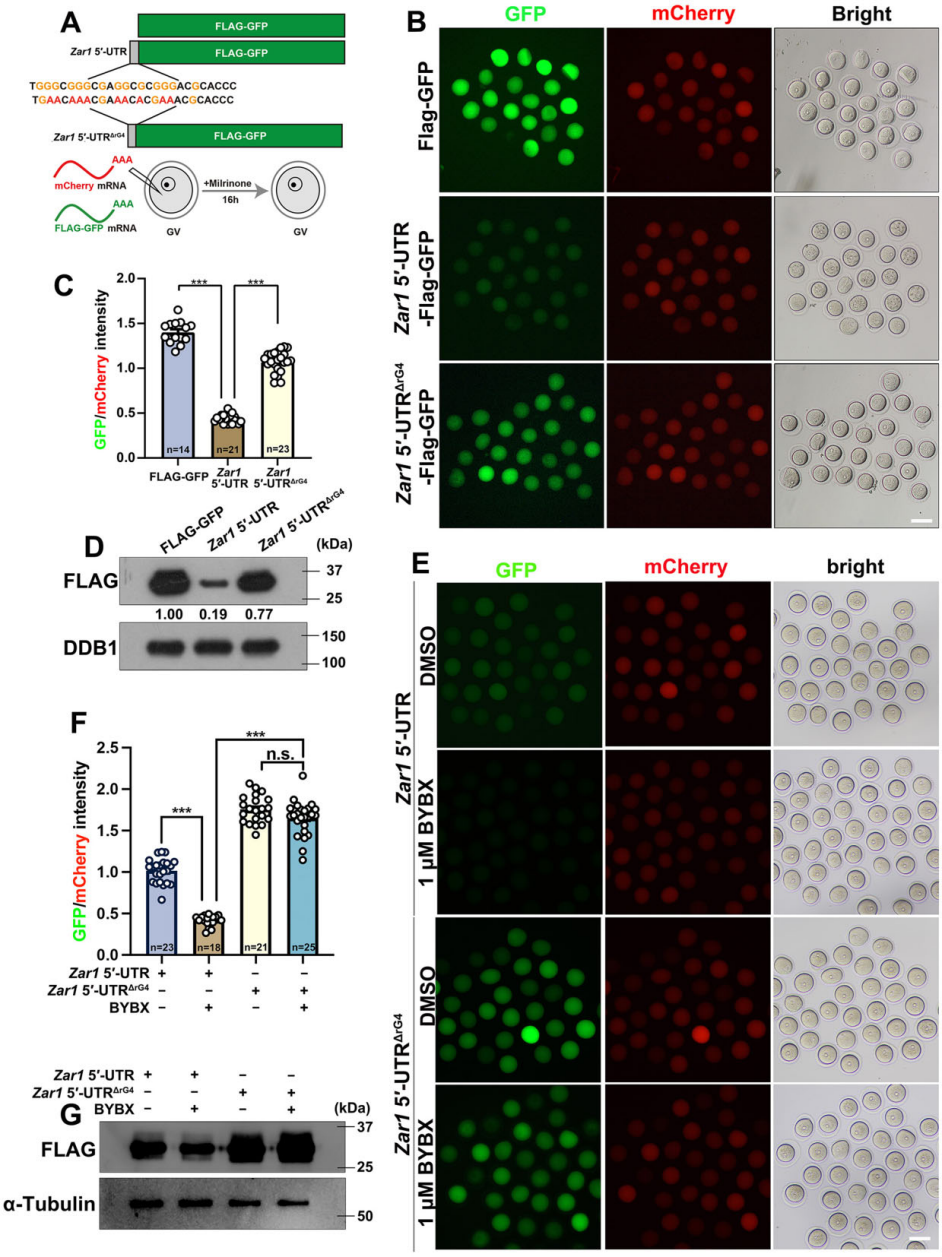
rG4s in *Zar1* 5′-UTRs disrupt translation in GV oocyte. (**A**) Illustration of the *Zar1* 5′-UTR reporter assay. (**B**) Fluorescence microscopy results showing the translational activity of the *Flag-Gfp* mRNAs with or without fusion with *Zar1* 5′-UTR or *Zar1* 5′-UTR^ΔrG4^ in GV-arrested (maintained by 2 μM milrinone) oocytes. (**C**) Quantification of the fluorescent signals in (**B**). (**D**) Western blot results using oocytes in (**B**). (**E**) Fluorescence microscopy results showing the translational activity of the *Flag-Gfp* mRNAs with *Zar1* 5′-UTR or *Zar1* 5′-UTR^ΔrG4^ in GV-arrested oocytes after DMSO and BYBX treatment. (**F**) Quantification of the fluorescent signals in (**E**). (**G**) Western blot results using the oocytes in (**E**). In C and F, error bars indicate SEM. ***P < 0.001 by two-tailed Student's t-test. The number of oocytes analyzed is indicated (n).

To verify the influences of rG4s in 5′-UTR on translational initiation of maternal mRNAs, we selected 5′-UTR of *Zar1* as an example, which had been verified to contain rG4s. In addition, the 5′-UTR of *Zar1* is only 28 bp, its structure is relatively simple, and it excludes the influences of other elements. We cloned the mouse *Zar1* 5′-UTR (WT and ΔrG4) into a GFP reporter plasmid, transcribed it into mRNA and polyadenylated *in vitro*, and microinjected it into GV oocytes. The *in vitro* polyadenylated transcript encoding mCherry was used as a positive control. Microinjected oocytes were further cultured for 16 h in the M16 medium containing milrinone, which repressed GVBD (Fig. 6A). After 16 hours, we detected stronger GFP signals in the oocytes microinjected *Flag-Gfp* mRNA without *Zar1* 5′-UTR fusion than those with *Zar1* 5′-UTR fusion. However, fusion with the *Zar1* 5′-UTR^ΔrG4^ did not repress the translation of *Flag-Gfp* mRNA (Fig. 6B and C). The mCherry signals were equal in the oocytes of the three groups. Western blotting using an anti-FLAG antibody showed a similar tendency to that of GFP fluorescence (Fig. 6D). These results indicated that the rG4s in the *Zar1* 5′-UTR directly impaired the translation.

Furthermore, we cultured oocytes injected with *Zar1* 5′-UTR and *Zar1* 5′-UTR^ΔrG4^ in medium containing DMSO or BYBX. GFP fluorescence (Fig. 6E and F) and FLAG western blot (Fig. 6G) results showed that the expression of the reporter protein driven by *Zar1* 5′-UTR was compromised in GV oocytes treated with BYBX. Similar defects were observed in oocytes treated with P67 (Supplementary Fig. S3A–C). The *Flag-Gfp* mRNA containing *Zar1* 5′-UTR^ΔrG4^ exhibited a much higher GFP signal compared to that containing *Zar1* 5′-UTR (Fig. 6E). More importantly, the expression of *Flag-Gfp* mRNA driven by *Zar1* 5′-UTR^ΔrG4^ showed no significant differences after BYBX treatment (Fig. 6E–G), confirming that BYBX impaired translation activity by stabilizing rG4s in the *Zar1* 5′-UTR.

### Balanced rG4s ensure translational activation of maternal transcripts during oocyte maturation

**Figure 7.**
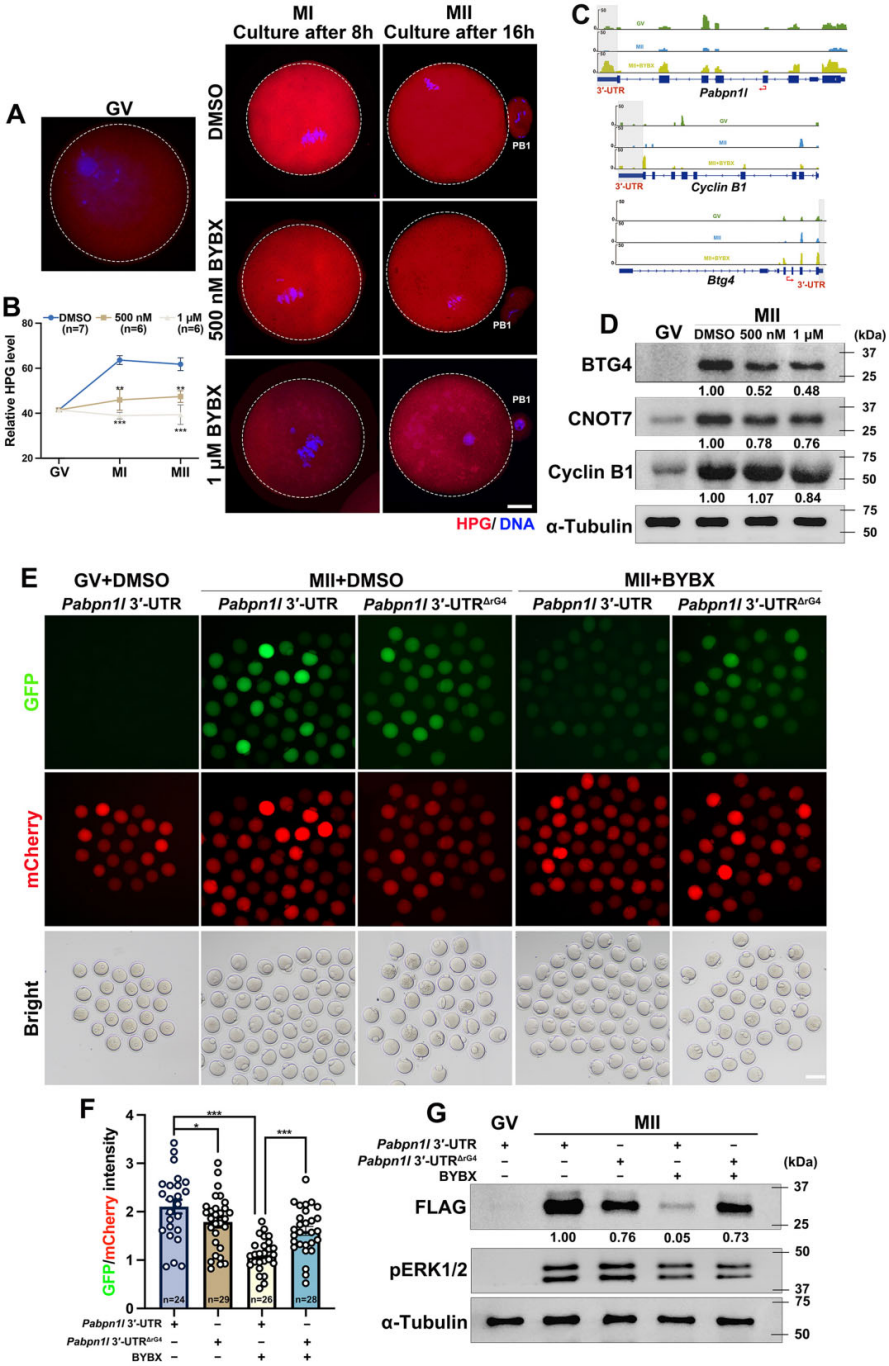
Impact of BYBX treatment on translational activation of maternal transcripts during oocyte maturation. (**A**-**B**) HPG fluorescent staining (**A**) and quantification (**B**) results showing the overall translation levels of oocytes cultured in medium with or without BYBX. Scale bar = 20 μm. (**C**) Snapshot of indicated genes showing accumulated rG4s in oocytes with or without BYBX treatment. (**D**) Western blot results showing the levels of BTG4, CNOT7, and CCNB1 in oocytes with or without BYBX treatment during *in vitro* maturation. (**E**-**G**) Fluorescence microscopy (**E**), quantification of the fluorescent signals (**F**), and western blot (**G**) results showing the translational activity of the *Flag-Gfp* mRNAs fused with *Pabpn1l* 3′-UTR or *Pabpn1l* 3′-UTR^ΔrG4^ in MII oocytes after DMSO and BYBX treatment. An *in vitro* transcribed and polyadenylated mCherry mRNA was co-microinjected as a positive control.

Translational activation of specific mRNAs is essential for the meiotic maturation of oocytes. After GVBD, many maternal mRNA are polyadenylated, and translational activation occurs. The influence of rG4s on the translatome suggests that it also affects meiotic-maturation-coupled translational activation in oocytes. L-HPG is a methionine analog that is incorporated into nascent proteins during protein synthesis. The incorporated HPG signals indicated the overall translational level. Oocytes were incubated in a medium containing HPG during oocyte maturation. Consistent with previous studies, oocytes at the GV stage were seen to have maintained low levels of translation, and the HPG signal was significantly elevated only after the resumption of meiosis (Fig. 7A). However, BYBX decreased nascent protein synthesis in oocytes at 8 or 16 h after culture (Fig. 7A and B), suggesting that rG4 accumulation represses translational activation in oocytes.

Successful translational activation of transcripts in oocytes depends on the recruitment of multiple RBPs to the 3′-UTR [66]. To explore whether the failure of the translational activation caused by BYBX was due to enrichment of rG4s in the 3′-UTR, we observed the expression patterns of transcripts that underwent translational activation during oocyte maturation. BTG4 is a meiotic cell cycle-coupled MZT licensing factor in mouse oocytes that triggers maternal mRNA decay by recruiting the CCR4–NOT catalytic subunit, CNOT7 or CNOT8, to actively translate mRNAs [6, 67]. Cyclin B1 promotes G2/M transition during oocyte meiosis [68]. PABPN1L participates in the regulation of mRNA translation by binding to the poly (A) tail [69]. The snapshots of these transcripts showed that the rG4 signals in the 3′-UTR significantly increased after BYBX treatment (Fig. 7C). The expression of BTG4, CNOT7, and Cyclin B1 was substantially attenuated (Fig. 7D), suggesting that the accumulation of rG4s disrupts the translational activation of important proteins during oocyte maturation.

We transcribed unpolyadenylated mRNA encoding *Flag-Gfp* fused with the *Pabpn1l* 3′-UTR and microinjected it into GV oocytes. The *in vitro* polyadenylated mCherry mRNA was co-injected as a positive control (Supplementary Fig. S3D). After 16 h of incubation, we detected weak GFP signals in GV-arrested oocytes and strong GFP signals in MII oocytes due to the 3′-UTR-driven translational activation. However, the translational activity of *Pabpn1l* 3′-UTR was repressed by BYBX treatment (Fig. 7E). A similar effect was observed in oocytes after treatment with P67 (Supplementary Fig. S3E–G). However, the expression of *Flag-Gfp* mRNA driven by *Pabpn1l* 3′-UTR^ΔrG4^ showed no significant differences after BYBX treatment (Fig. 7E and F). The mCherry signals were consistent across all four groups. Western blotting using an anti-FLAG antibody showed a similar trend to that of GFP fluorescence (Fig. 7G). Since the destruction of the G-rich sequence in the 3′-UTR may affect some RBPs binding to RNA, we constructed the *Pabpn1l* 3′-UTR^ΔrG4^ reporter, which minimally disrupts the G-rich sequence but is insufficient to form an rG4 structure. However, the expression of mRNA containing *Pabpn1l* 3′-UTR^ΔrG4^ was partially reduced. In addition, we also microinjected mRNAs encoding *Flag-Gfp* fused with *Btg4* and *Ccnb1* 3′-UTRs. Similarly, the expressions were repressed after BYBX treatment (Supplementary Fig. S3G-K). Collectively, these results indicate that rG4s stabilized by BYBX in 3′-UTRs impair the translational activation of maternal transcripts during oocyte maturation.

### DHX36 is a rG4 resolvase and reverses the pharmacological effects of BYBX in mouse oocytes

**Figure 8.**
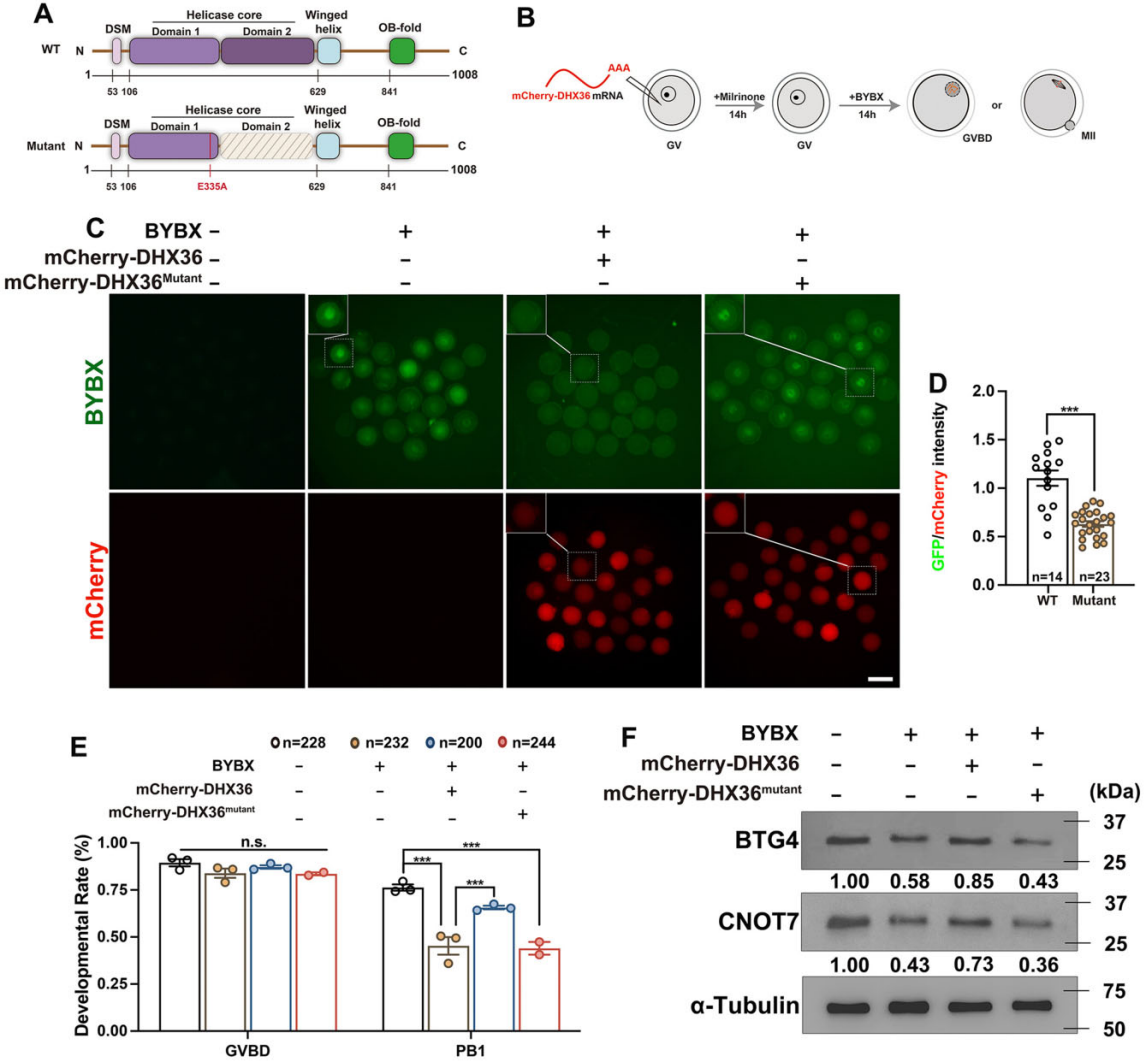
DHX36 partially attenuates the effects of BYBX in mouse oocytes. (**A**) Domain structure of WT and mutant DHX36. E335 is a residue essential for helicase activity. Domain 1 and Domain 2 represent the helicase cores. (**B**) Illustration of the *Dhx36*-*mCherry* mRNA microinjections. (**C**) BYBX fluorescence results showing rG4 levels in MII oocytes overexpressing DHX36 (WT and mutant). Scale bar, 100 μm. (**D**) Relative intensity of the BYBX signal in (**C**) after normalization by the mCherry signal in the same oocyte. (**E**) Rates of GVBD and PB1 emission in oocytes after BYBX treatment and DHX36 overexpression. (**F**) Western blot results showing the levels of BTG4 and CNOT7 in MII oocytes after BYBX treatment and DHX36 overexpression. Total proteins from 100 oocytes were loaded in each lane. α-tubulin was used as a loading control. The protein band intensity was analyzed using ImageJ and labeled under the corresponding bands. In (**D**) and (**E**), error bars indicate SEM. ***P< 0.001 by two-tailed Student's t-test. n.s.: non-significant. The number of oocytes analyzed is indicated (n).

The DEAH/RHA helicase DHX36 plays a crucial role in the removal of rG4s linked to cellular RNA quadruplexes and AU-rich RNA elements. DHX36 contains a superfamily 2 helicase core and several auxiliary domains conserved across species (Fig. 8A). The helicase core of DHX36, particularly the E335 residue, is essential for the unfolding of rG4s [70].

To further explore whether DHX36 rescues BYBX-induced rG4 damage during oocyte maturation, we cloned the full-length mouse DHX36 and both domain 1 and domain 2 mutations to fully inactivate DHX36 into an mCherry-tagged expression plasmid, transcribed them into mRNAs, and microinjected them into GV oocytes. Microinjected oocytes were cultured for 14 h in medium with milrinone to overexpress DHX36, followed by 14 h of culture in medium with or without BYBX (Fig. 8B). As an RNA-specific fluorescent ligand of rG4, BYBX induces rG4 accumulation and generates green fluorescence signals upon binding. After 14 h of incubation with BYBX, strong BYBX signals were detected in the oocytes. However, overexpression of DHX36, but not DHX36^mutant^, substantially attenuated the BYBX fluorescence. mCherry signals were equal in the oocytes of both groups (Fig. 8C and D).

Notably, the PB1 emission rate of oocytes was partially rescued by DHX36 overexpression in the BYBX-treated oocytes (Fig. 8E). Western blot results showed that DHX36 overexpression enhanced the expression of BTG4 and CNOT7, which was significantly inhibited after BYBX treatment (Fig. 8F). Taken together, these data indicated that DHX36 can counteract the effect of BYBX in stabilizing rG4s, restoring the dynamic equilibrium of rG4s in oocytes, and partially rescuing oocyte meiotic maturation.

## Discussion

Oocytes have special advantages and properties for studying rG4s. Oocytes exhibit vigorous transcriptional activity during growth, and they store large amounts of maternal mRNAs. After reaching the fully grown state, the oocytes undergo transcriptional repression, and the genome does not resume active transcription until the 2-cell stage after fertilization. During this period, oocyte development and fertilization activities depend completely on the maternal mRNAs synthesized in the early stages. This special transcriptional silencing stage is beneficial for studying the role of rG4 in post-transcriptional regulation.

**Figure 9.**
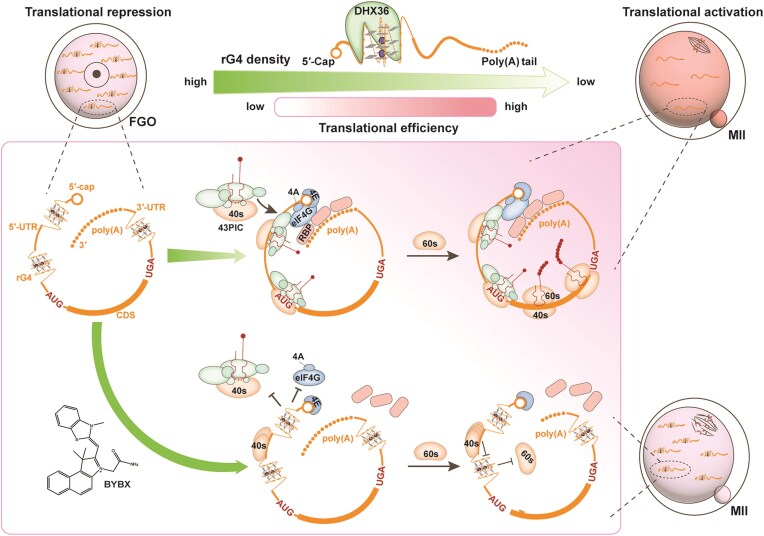
Summary of rG4s distribution and regulation during oocyte maturation. Maternal mRNAs are translationally repressed due to the enrichment of rG4s in GV oocytes. These rG4s are recognized and removed by DHX36 and trigger translational activation during meiotic maturation. After BYBX treatment, the accumulation of rG4s in the 5′-UTR prevents eIF4A, 4G, and 43PIC from binding to the RNA. Meanwhile, rG4s hinder the scanning of the 5′-UTR by the 40s ribosome, preventing the correct assembly with the 60s ribosome, ultimately disrupting the initiation of translation. rG4s can also disrupt translational activation of maternal mRNAs by impairing the binding of RBPs to the 3′-UTRs. The accumulation of rG4s reduces the overall translation levels, resulting in a decrease in the oocyte quality. Therefore, the destabilization and unfolding of rG4s are crucial factors for specific translation activation after the resumption of meiosis in oocytes.

Oocytes resume meiosis after GVBD, and the stored transcripts undergo translational activation. Simultaneously, we observed enriched UTR localization of rG4 in GV stage-arrested oocytes and meiotic maturation-coupled extensive rG4 removal in the maternal transcriptome. Furthermore, rG4s in both 5′- and 3′-UTRs hindered translation of selective maternal transcripts, and rG4 stabilization by BYBX repressed translations at the whole transcriptome level. Therefore, based on the function of rG4s and their decreasing distributions from the GV-to-MII transition, we hypothesized that before meiosis resumption, large number of rG4s in oocytes are necessary to maintain the translatome at a low level, and DHX36-mediated rG4 removal during meiotic maturation facilitates translational activation of key maternal factors that are required for successful MZT (Fig. 9). Therefore, during oocyte maturation, dynamic changes in mRNA translation efficiencies require not only the trans-regulation of key RBPs but also the *cis-*regulation of specific RNA structures, such as rG4s. Conceptually, these investigations not only revealed the regulatory network of rG4 in oocyte maturation, but they also provided new targets for the clinical diagnosis and treatment of infertility in assisted reproduction.

To elucidate the physiological importance of rG4 removal during oocyte maturation, we stabilized rG4s using the fluorescent probe, BYBX. In previous studies, small-molecule G4 ligands such as pyridostatin [71] stabilized both dG4s and rG4s in cells, and CYTO-4C [62] specifically stabilized the dG4 structure. Similarly, depletion of G4 helicases in cells causes simultaneous accumulation of dG4s and rG4s. In this study, although we investigated the function of DHX36 in oocytes, this G4 helicase is only expressed or inhibited in fully grown transcriptionally silent oocytes. Therefore, these approaches can effectively exclude the influence of the dG4 structure on experimental results.

With the continuous progress in high-throughput analysis technology, various detection methods for rG4s have emerged. Previously developed rG4-seq techniques can detect rG4s by exploiting the properties of G4s stabilized in K^+^ solutions and unstable in Li^+^ solutions [21]. However, the presence of K^+^ promotes the formation of G4 structures, resulting in the inability to truly display normal intracellular rG4 distributions. To solve this problem, researchers developed SHALIPE-seq and other methods [29, 44]. However, for low-abundance samples, such as oocytes, the number of cells required for these methods is too large, and the procedure is complicated. In this study, we have established a low-input, high-sensitivity G4-LACE-seq method for oocytes using LACE-seq technology and a G4-specific BG4 monoclonal antibody. In the LACE-seq approach, the BG4 antibody detects rG4 by steric hindrance rather than by its binding affinity and immunoprecipitation with rG4s, as previously reported. Moreover, the *in vitro* RNA transcription further increased the RNA yield for subsequent sequencing. Therefore, the G4-LACE-seq method allows for low input, high sensitivity, and single-nucleotide precision for rG4 detection in mouse oocytes. Using this technique, we determined the dynamic distribution of rG4s during oocyte maturation. The precision and fidelity of this method are verified by: 1) the enrichment of guanosine residues in the consensus rG4 motives based on G4-LACE-seq results; 2) this significant increase in rG4 peaks detected after BYBX treatment as expected; 3) deletion of the detected rG4s in Zar1 5′-UTR abolished the binding with BYBX as well as the repressive effect on translation. The development of the G4-LACE-seq technology provides a new approach for the detection of rG4 in low-input cells and facilitates efficient and in-depth studies of rG4 in cells.

Previous studies have demonstrated the role of rG4s in RNA translation [26], degradation [27, 28], and also the regulation of rG4-binding proteins [33–35], mostly in cultured cell lines. However, more attention should be paid to the physiological functions of rG4 regulation. RNAs interact with a broad spectrum of RBPs that can regulate RNA stability, splicing, translation, and localization. However, the effects of rG4s on RBPs have not yet been sufficiently investigated in previous studies. We optimized the published RBPs detection method and made the first effort to assess the effect of rG4 on RBPs binding at the whole-proteome level. Although the RBPs observed in 293T cells may not fully reflect the situation in oocytes, and some oocyte-specific proteins may be missed, the translation initiation complex and ribosomal large and small subunits were conserved between 293T cells and oocytes. After BYBX treatment, the binding of maternal transcripts to RBPs related to ribosomal structural components, translation initiation complexes, mRNA processing, and cytoplasmic stress granule assembly was significantly reduced. This suggests that rG4s extensively impair RBP binding and influence multiple aspects of RNA metabolism during oocyte maturation.

Through translation-related reporter assays and RBP analyses, we proposed that rG4s present in 5′-UTR may have a greater defect than that in 3′-UTR. However, the previous studies in mouse and human oocytes mainly focused on the regulatory effects of different elements of 3′-UTR on mRNA tailing and translation, while there was little focus on the regulatory effects of 5′-UTR elements on translation. Our study illustrated the important role of G4 unwinding on 5′-UTRs in mouse oocyte translation. The results called attention to the fact that more elements and RNA secondary structures in 5′-UTR should be investigated in future research, especially their impacts on maternal mRNA translation.

## Supplementary Material

gkaf067_Supplemental_File

## Data Availability

The data were deposited in the NCBI Gene Expression Omnibus database under the accession codes LACE-seq: GSE262730, Ribo-lite: GSE262943, and RNA-seq: GSE262608.
