# Supplementary material for: RNA G-quadruplex removal promotes a translational switch after meiosis resumption
Source: Nucleic Acids Res. 2025 Apr 30;53(8):gkaf067. doi: 10.1093/nar/gkaf067 (PMC12041855; doi:10.1093/nar/gkaf067)
Supplement: gkaf067_Supplemental_File [file gkaf067_supplemental_file.pdf]

Supplementary Figures

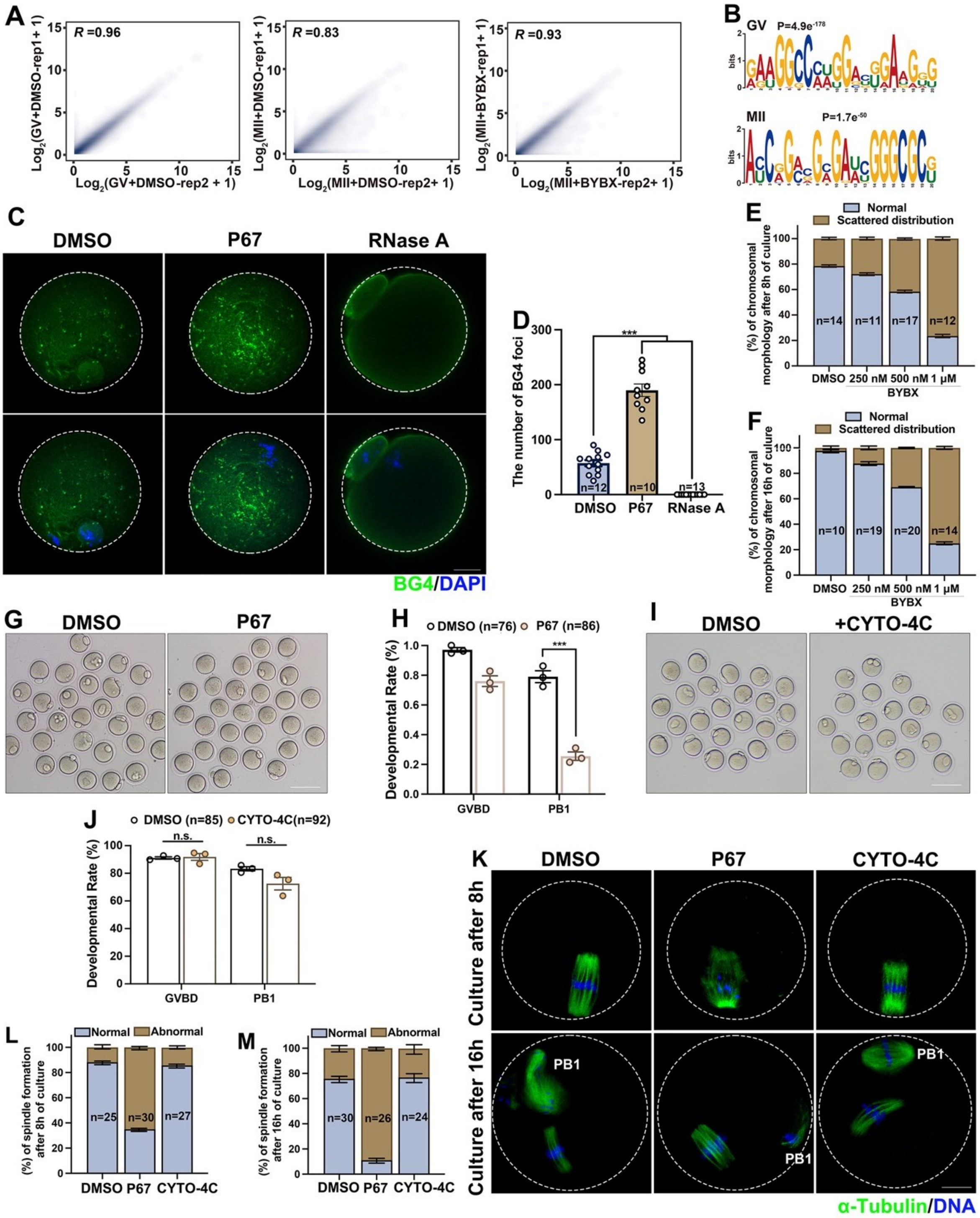

Figure S1: LACE-seq analyses in mouse oocytes during meiotic maturation. **A**: Scatter plot showing the correlation of G4-LACE-seq results between biological replicates. **B**: Sequence motifs of rG4s identified by BG4-LACE-seq in GV and MII oocytes. $P = 4.9e^{-178}$ and $P = 1.7e^{-50}$ by two-tailed Student's t test. **C**: Confocal microscopic images of DAPI (blue) and BG4

(green) immunofluorescence in MII oocytes after P67 and RNase A treatment. Scale bar = 20 μm. **D:** The number of BG4 foci in MII oocytes after P67 and RNase A treatment. **E-F**: Percentage (%) of chromosomal morphology in oocytes cultured for 8 (**E**) and 16 h (**F**). **G-H:** Representative images of oocytes at 16 h of *in vitro* maturation culture with 1 μM P67 (**G**) and 1 μM CYTO-4C (**H**). Scale bar = 100 μm. **I-J**: Rates of GVBD and PB1 emission in the oocytes cultured with 1 μM P67 (**I**) and 1 μM CYTO-4C (**J**). **K**: Confocal microscopy results showing spindle assembly in the oocytes cultured for 8 h and 16 h after P67 and CYTO-4C treatment. Scale bar = 20 μm. **L-M**: Percentage (%) of normal spindles in oocytes after 8 (**L**) and 16 h (**M**) culture in medium containing DMSO or BYBX. In **D-F, I-J,** and **L-M**, the error bars indicate SEM. ***$P < 0.001$ by two-tailed Student's t-test. The number of oocytes analyzed is indicated (n).

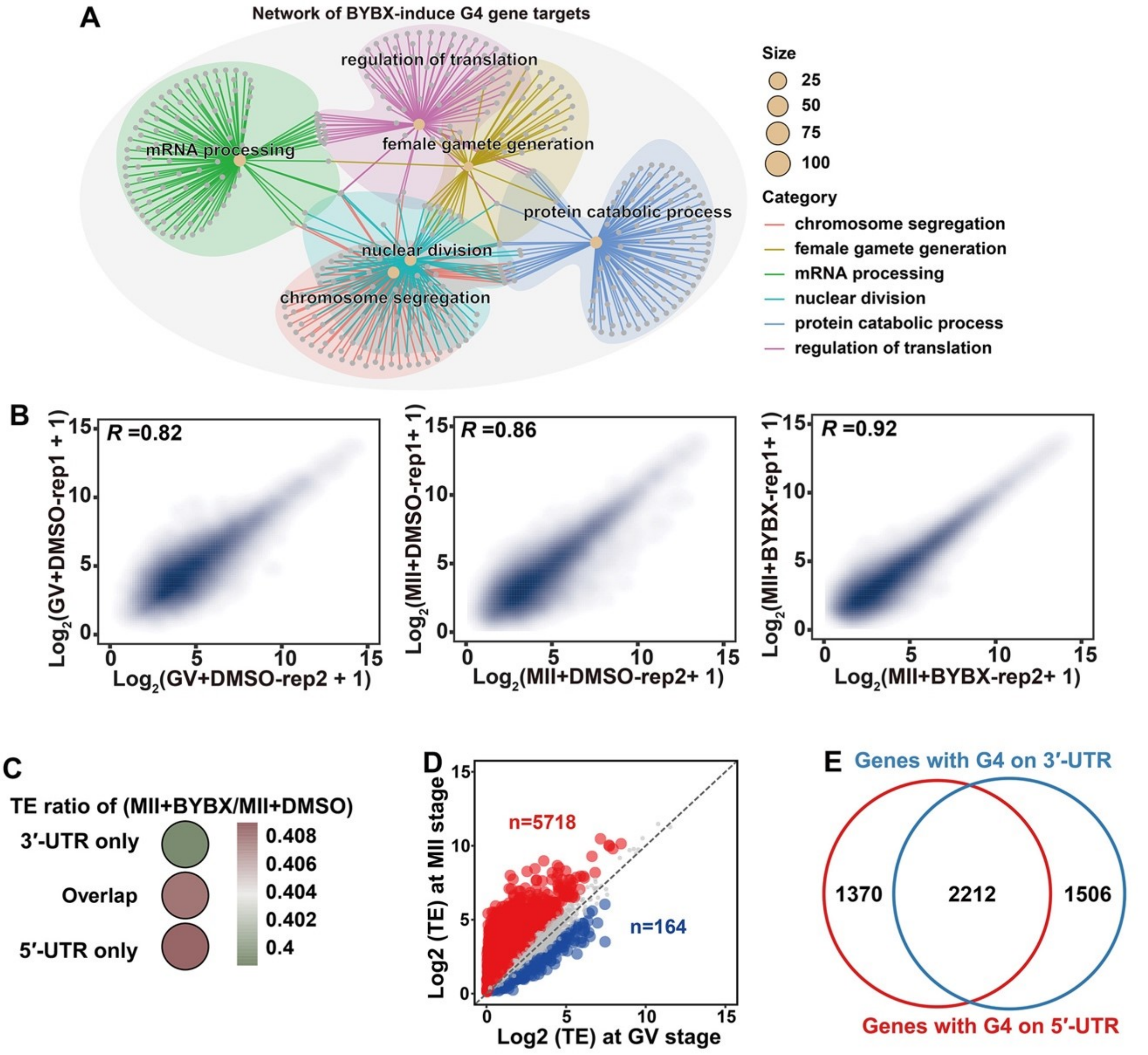

Figure S2: Ribo-seq analyses of mRNA translation efficiency during oocyte maturation. **A**: Network analysis of the enriched GO terms in transcripts containing BYBX-induced rG4s. B: Scatter plot showing the correlation of Ribo-lite results between biological replicates in oocytes. **C**: BYBX-induced TE changes of transcripts containing rG4s in their 3′-UTRs, 5′-UTRs, or both in MII oocytes. **D**: Scatter plot showing TE changes of transcripts in GV and MII oocytes. Transcripts with TEs decreased or increased more than 2-fold in MII oocytes were highlighted with blue or red, respectively. **E**: Venn plot showing the overlap of transcripts with rG4s in 3′- and 5′-UTRs.

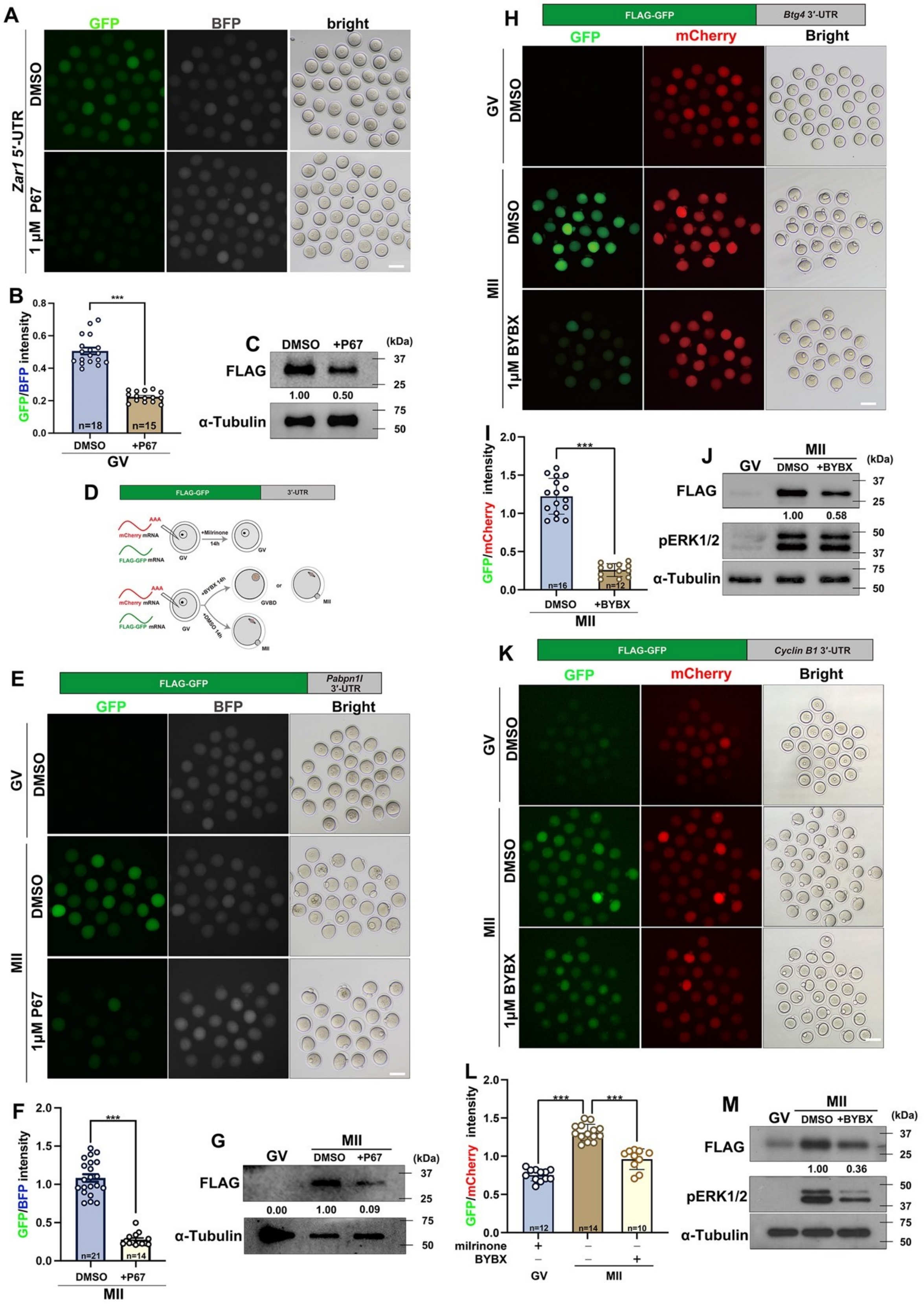

Figure S3: Translational activities of mRNAs with rG4s in the UTRs. A: Fluorescence microscopy results showing the translational activity of the *Flag-Gfp* mRNAs with *Zar1* 5′-

UTR in GV-arrested oocytes with DMSO or P67 treatment. **B**: Quantification of the fluorescent signals in (**A**). **C**: Western blot results using oocytes from (**A**). **D**: Schematic diagram of the oocyte microinjection with 3′-UTR-fused *Flag-Gfp* mRNAs. **E**: Fluorescence microscopy results showing the translational activity of the *Flag-Gfp* mRNAs with *Pabpn1l* 3′-UTR in GV-arrested oocytes and MII oocytes with DMSO or P67 treatment. **F**: Quantification of the fluorescent signals in (**E**). **G**: Western blot results using oocytes from (**E**). **H-J**: Fluorescence microscopy (**H**), signal quantification (**I**), and western blot (**J**) results showing the translational activity of the *Flag-Gfp* mRNAs fused with *Btg4* 3′-UTR in GV and MII oocytes. **K-M**: Fluorescence microscopy (**K**), signal quantification (**L**), and western blot (**M**) results showing the translational activity of the *Flag-Gfp* mRNAs fused with *Ccnb1* 3′-UTR in GV and MII oocytes. An *in vitro* transcribed and polyadenylated mCherry mRNA was co-microinjected as a positive control. In **B, F, I,** and **L**, the error bars indicate SEM. ***P < 0.001 by two-tailed Student's t-test. The number of oocytes analyzed is indicated (n).

Table S1: Spearman correlation coefficients of Lace-seq among among GV oocytes and MII oocytes after DMSO and BYBX treatment.

	GV-rep1	GV-rep2	MII-rep1	MII-rep2	MII+BYBX-rep1	MII+BYBX-rep2	IgG
GV-rep1	1.00	0.96	0.60	0.71	0.69	0.75	0.37
GV-rep2	0.96	1.00	0.60	0.72	0.69	0.76	0.38
MII-rep1	0.60	0.60	1.00	0.82	0.84	0.71	0.34
MII-rep2	0.71	0.72	0.82	1.00	0.77	0.85	0.37
MII+BYBX-rep1	0.69	0.69	0.84	0.77	1.00	0.89	0.35
MII+BYBX-rep2	0.75	0.76	0.71	0.85	0.89	1.00	0.38
IgG	0.37	0.38	0.34	0.37	0.35	0.38	1.00

Table S2. Antibody information

**Protein name**	**Manufacture (catalogue number)**	**Applications (working dilution)**
BG4	Absolute antibody (Ab00174-1.1)	IF (1:250)
H3K4me3	Cell Signaling (9727)	IF (1:500)
FITC-α-Tubulin	Sigma (F2168)	IF (1:500) WB (1:2000)
BTG4	Abcam (ab206914)	WB (1:1000)
CNOT7	Abcam (ab195587)	WB (1:1000)

CCNB1	Cell Signaling (4138)	WB (1:1000)
FLAG	Sigma (F3165)	WB (1:3000)
pERK1/2	Cell Signaling (9101)	WB (1:1000)
DDB1	Epitomics (3821-1)	WB (1:10000)

Table S3: Spearman correlation coefficients of ribo-lite among among GV oocytes and MII oocytes after DMSO and BYBX treatment.

	GV-rep1	GV-rep2	MII-rep1	MII-rep2	MII+BYBX-rep1	MII+BYBX-rep2
GV-rep1	1.00	0.97	0.40	0.37	0.51	0.52
GV-rep2	0.97	1.00	0.45	0.41	0.53	0.53
MII-rep1	0.40	0.45	1.00	0.90	0.87	0.87
MII-rep2	0.37	0.41	0.90	1.00	0.96	0.96
MII+BYBX-rep1	0.51	0.53	0.87	0.96	1.00	1.00
MII+BYBX-rep2	0.52	0.53	0.87	0.96	1.00	1.00

Table S4: Spearman correlation coefficients of RNA-seq among among GV oocytes and MII oocytes after DMSO and BYBX treatment.

	GV-rep1	GV-rep2	MII-rep1	MII-rep2	MII-rep3	MII+BYBX-rep1	MII+BYBX-rep2
GV-rep1	1.00	0.97	0.55	0.54	0.55	0.84	0.90
GV-rep2	0.97	1.00	0.53	0.55	0.56	0.87	0.89
MII-rep1	0.55	0.53	1.00	0.97	0.97	0.76	0.78
MII-rep2	0.54	0.55	0.97	1.00	0.98	0.80	0.77
MII-rep3	0.55	0.56	0.97	0.98	1.00	0.81	0.79
MII+BYBX-rep1	0.84	0.87	0.76	0.80	0.81	1.00	0.94
MII+BYBX-rep2	0.90	0.89	0.78	0.77	0.79	0.94	1.00
